# Targeting of apoptotic pathways by SMAC or BH3 mimetics distinctly sensitizes paclitaxel-resistant triple negative breast cancer cells

**DOI:** 10.18632/oncotarget.15125

**Published:** 2017-02-06

**Authors:** Effrosini G. Panayotopoulou, Anna-Katharina Müller, Melanie Börries, Hauke Busch, Guohong Hu, Sima Lev

**Affiliations:** ^1^ Molecular Cell Biology Department, Weizmann Institute of Science, Rehovot 76100, Israel; ^2^ Institute of Molecular Medicine and Cell Research (IMMZ), Albert Ludwigs-University, 79104 Freiburg, Germany; ^3^ Shanghai Institutes for Biological Sciences, Chinese Academy of Sciences, Shanghai 200031, China

**Keywords:** triple negative breast cancer, paclitaxel, resistance, SMAC mimetics, high-throughput screen

## Abstract

Standard chemotherapy is the only systemic treatment for triple-negative breast cancer (TNBC), and despite the good initial response, resistance remains a major therapeutic obstacle. Here, we employed a High-Throughput Screen to identify targeted therapies that overcome chemoresistance in TNBC. We applied short-term paclitaxel treatment and screened 320 small-molecule inhibitors of known targets to identify drugs that preferentially and efficiently target paclitaxel-treated TNBC cells. Among these compounds the SMAC mimetics (BV6, Birinapant) and BH3-mimetics (ABT-737/263) were recognized as potent targeted therapy for multiple paclitaxel-residual TNBC cell lines. However, acquired paclitaxel resistance through repeated paclitaxel pulses result in desensitization to BV6, but not to ABT-263, suggesting that short- and long-term paclitaxel resistance are mediated by distinct mechanisms. Gene expression profiling of paclitaxel-residual, -resistant and naïve MDA-MB-231 cells demonstrated that paclitaxel-residual, as opposed to -resistant cells, were characterized by an apoptotic signature, with downregulation of anti-apoptotic genes (*BCL2, BIRC5*), induction of apoptosis inducers (*IL24, PDCD4*), and enrichment of TNFα/NF-κB pathway, including upregulation of *TNFSF15*, coupled with cell-cycle arrest. *BIRC5* and *FOXM1* downregulation and *IL24* induction was also evident in breast cancer patient datasets following taxane treatment. Exposure of naïve or paclitaxel-resistant cells to supernatants of paclitaxel-residual cells sensitized them to BV6, and treatment with TNFα enhanced BV6 potency, suggesting that sensitization to BV6 is mediated, at least partially, by secreted factor(s). Our results suggest that administration of SMAC or BH3 mimetics following short-term paclitaxel treatment could be an effective therapeutic strategy for TNBC, while only BH3-mimetics could effectively overcome long-term paclitaxel resistance.

## INTRODUCTION

Triple negative breast cancer (TNBC) is defined by the absence of estrogen receptor (ER), progesterone receptor (PR) and HER2 amplification and constitutes an exceedingly heterogeneous group of breast cancers, generally stratified into six distinct molecular subtypes including two basal-like subtypes (BL1 and BL2) in addition to immunomodulatory (IM), mesenchymal (M), mesenchymal stem–like (MSL), and luminal androgen receptor (LAR) subtypes [1, 2]. Epidemiologically, TNBC accounts for approximately 20% of all breast cancers and is characterized by high mitotic indices, high rates of metastasis and poor prognosis [1]. The currently available treatment options for TNBC management rely entirely on sequential or concurrent administration of conventional chemotherapeutic agents (mainly anthracycline/taxane-based regimens) [3]. However, chemoresistance is a major clinical drawback and TNBC patients have the highest recurrence rate and remarkably reduced survival within the first 3–5 years after primary treatment [4, 5].

Among the different chemotherapeutic agents, paclitaxel is commonly used in clinical practice to treat TNBC patients. In fact, the taxanes paclitaxel (Taxol) and docetaxel (Taxotere) were the first microtubule-stabilizing agents approved for use in solid tumors [6]. They have demonstrated activity, either as single agents or in combination with other chemotherapeutic or target-specific drugs, against a broad spectrum of malignancies, including breast cancer [7]. However, the clinical success of taxanes has been compromised by the emergence of drug resistance, as well as numerous side-effects, including neutropenia and neurotoxicity [8]. Acquired taxane resistance can be mediated by multiple mechanisms that affect drug transport or metabolism, modify tubulin structure, or perturb signal transduction pathways, including apoptosis-related pathways [6]. Drug resistance impedes the initial treatment, as well as the adjuvant setting and has been estimated to cause treatment failure in > 90% of patients with metastatic disease [9].

Paclitaxel targets microtubules to interfere with the mitotic spindle, resulting in cell cycle arrest and ultimately apoptosis, in a microtubule dysfunction-dependent and -independent manner [6]. Although paclitaxel eliminates most tumor cells, the mechanisms leading to resistance in the case of residual cancer cells are unclear [10].

Currently, more than 100 ongoing clinical trials implement paclitaxel in combination with sequential or concurrent administration of either chemotherapeutic agents or inhibitors of specific molecular targets (clinicaltrials.gov). In clinic, paclitaxel and anthracyclines (doxorubicin, epirubicin) have been introduced as second and third generation regimens for treating low/moderate and high-risk breast cancer disease, respectively [3, 11]. Due to the pharmacokinetic interaction of paclitaxel/doxorubicin and the accompanied increased cardiotoxicity, the drugs are often applied sequentially [3].

Since chemotherapy remains the mainstay treatment for TNBC and resistance is a significant hurdle in clinical practice, identification of therapeutic strategies to overcome chemoresistant disease is a major challenge. Here, we employed a high-throughput screen (HTS) of paclitaxel-treated cells, utilizing small molecule inhibitors with known molecular targets to identify drugs that preferentially and efficiently kill paclitaxel-residual TNBC cells, using a protocol of four days treatment with paclitaxel following by four days recovery in drug-free medium. Among the 320 compounds that were screened, we discovered that SMAC mimetics, which inhibit IAPs (Inhibitors of Apoptosis Proteins), and BH3 mimetics which target BCL-2 family members, effectively, potently and preferentially target TNBC cells that have escaped short-term paclitaxel treatment. However, adaptive paclitaxel-resistance, which was established by long-term exposure to paclitaxel, employing repeated cycles of drug-pulse/recovery was accompanied by resistance to SMAC mimetics, while the efficacy of BH3 mimetics was sustained. Gene expression profiling using Affymetrix microarray analysis, showed that paclitaxel-residual cells are characterized by upregulation of TNFα/NF-κB signaling coupled to G2/M cell cycle arrest, as well as upregulation of apoptotic (*IL24*, *PDCD4*) and downregulation of anti-apoptotic (*BIRC5, BCL2*) genes expression. This profile was reverted in paclitaxel-resistant cells. Furthermore, supernatants from paclitaxel-residual cells, as well as exogenous addition of TNFα and IL24, re-sensitized paclitaxel-resistant cells to the IAP inhibitor BV6, suggesting that sensitivity to apoptosis-inducing drugs following short-term paclitaxel treatment is mediated, at least in part, by secreted factor(s). We propose that targeting of different apoptotic pathways could distinctly affect the therapeutic response of TNBC patients following short- or long-term paclitaxel administration, and thus could provide effective regimens for different subsets of paclitaxel-treated patients.

## RESULTS

### Screening for small molecule inhibitors that preferentially and effectively target paclitaxel-residual TNBC cells

Although chemotherapy is the current treatment for most TNBC patients [5], the high frequency of recurrent disease and drug resistance strongly suggests that chemotherapy alone is not sufficient and combination therapy is required. Hence, we established a HTS employing a short-term paclitaxel treatment protocol to identify targeted therapies that eliminate paclitaxel-residual TNBC cells, using a small-molecule library of 320 compounds, of which some are FDA approved. The library consists of clinically relevant drugs, including tyrosine kinase inhibitors, proteasome and heat shock protein inhibitors, cell cycle inhibitors, DNA damage checkpoint inhibitors, pro-apoptotic drugs and anti-apoptosis inhibitors, chemotherapeutic agents and antimetabolites (The complete list is shown in Supplementary Table 1). These compounds target key signaling pathways and cellular effectors implicated in cancer initiation and progression (kinases, phosphatases, transporters, metabolic modulators, etc.). The screen was carried out using MDA-MB-231 cells and a protocol of four days treatment with paclitaxel followed by four days recovery in drug-free medium as previously described [12]. We have chosen paclitaxel as drug of interest, as it is commonly administered in clinical practice to treat TNBC patients [3] and selected the MDA-MB-231 cells as a representative line [13], along with six additional TNBC cell lines (SUM159T, BT549, HCC1143, HCC38, HCC1937, MDA-MB-468), which were further characterized throughout the study. More information on these TNBC cell lines is included in Supplementary Table 2.

**Figure 1:**
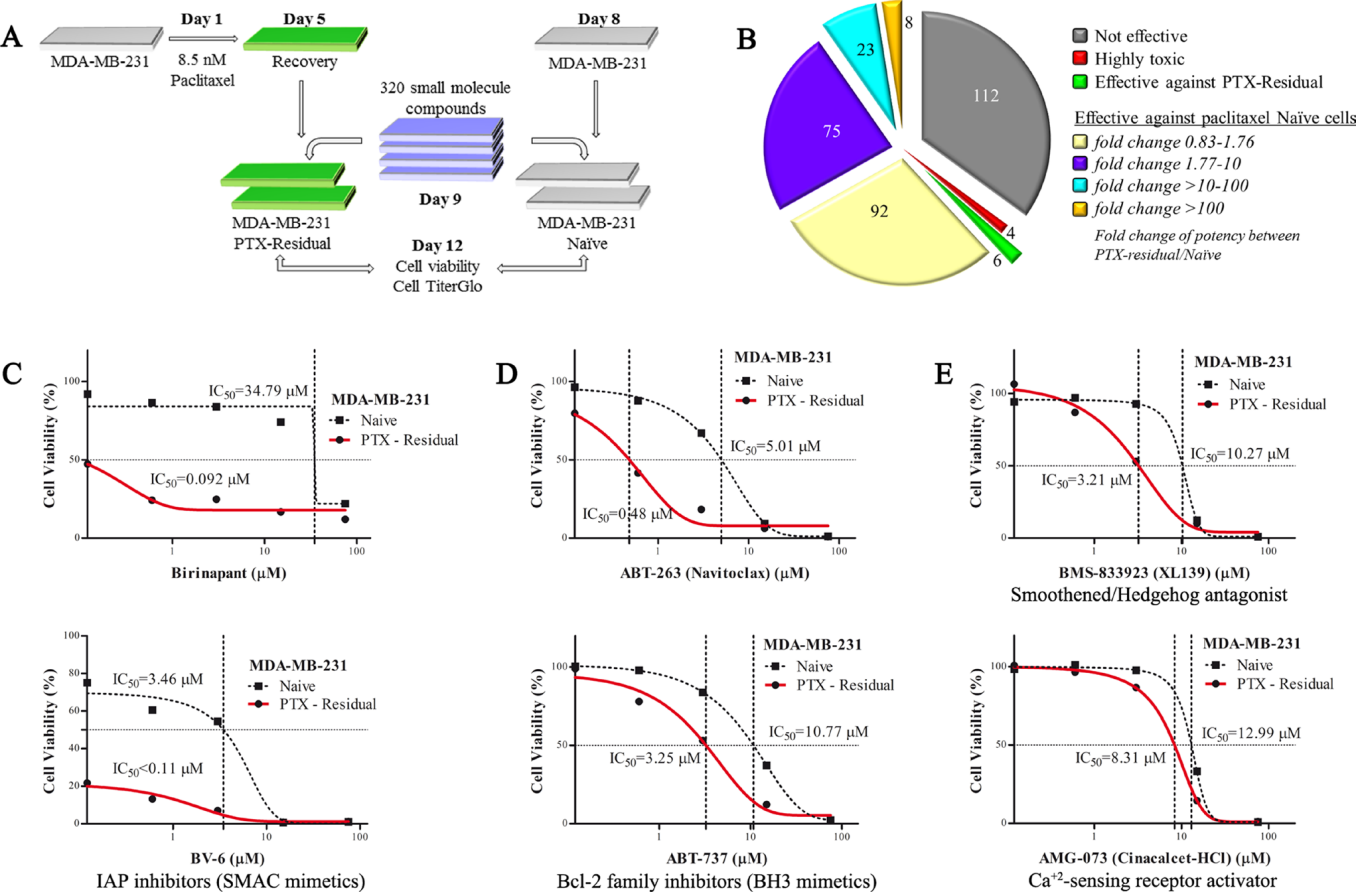
A High Throughput Screen (HTS) to identify effective compounds against paclitaxel-residual MDA-MB-231 cells (**A**) Cartoon of the HTS workflow. Day 0: seeding of 4 × 10^3^ MDA-MB-231 cells in 384-well white opaque TC plates in 40 μl of growth medium. Day 1: addition of paclitaxel at 5× concentration (final concentration, 8.5 nM) by the GNF instrument, followed by incubation for 96 h. Day 5: recovery in drug-free medium for 96 h utilizing robotic station (Biotek dispenser/Liconic incubator/BRAVO robot). Day 8: seeding of 1.5 × 10^3^ paclitaxel-naïve cells. Day 9: addition of the library of small molecule compounds by Echo transfer for 72 h in 5 serial dilutions (120 nM-75 μM and 16 nM-10 μM where appropriate) in triplicates. Day 12: Assessment of cell viability by CellTiter Glo luminescent cell viability assay, followed by automatic reading of the luminescent signal (Liconic incubator/BRAVO robot/PheraStar reader). (**B**) Graphical summary of the results of the HTS. Out of the 208 small molecule inhibitors that were effective against MDA-MB-231 cells, only 6 were selectively potent against paclitaxel-residual cells (green). Among the compounds that were more effective against paclitaxel-naïve cells (*N* = 198), treatment with paclitaxel caused variable increase of the IC_50_ as indicated. Notably, for 23 compounds the fold-increase of IC_50_ was 10–100, whereas for 8 compounds the IC_50_ was increased above 100-fold. (**C**–**E**) Effective small molecule inhibitors against paclitaxel-residual MDA-MB-231 cells. The small molecule inhibitors belong to two main categories, namely SMAC mimetics (C) and BCL-2 family inhibitors (BCL-XL, BCL-2, BCL-w) (D). Decrease in the viability of the paclitaxel-residual compared to parental paclitaxel-naïve cells was also observed following treatment with the SMO/HH pathway antagonist BMS-833923 (XL139) and the CaSR activator AMG-073 HCl (Cinacalcet hydrochloride) (E). PTX: Paclitaxel.

The screen included paclitaxel-treated and –naive untreated cells, with the ultimate goal to identify compounds that effectively and preferentially target paclitaxel-residual cells and not the parental naïve cells, and thus exhibit low toxicity and high efficacy. The HTS workflow is described in the Material and Methods and in Figure 1A, while the results are summarized in Figure 1B. As seen, among the 320 compounds that were screened, over one third (*N* = 112) were ineffective against either paclitaxel-residual or -naïve MDA-MB-231 cells in the applied concentrations range, while 198 compounds were more toxic for the naïve compared to the paclitaxel-residual cells. Four compounds were highly toxic for both paclitaxel-residual and –naïve cells, whereas six compounds (Birinapant, BV6, ABT-263/737, BMS833923 and AMG-073) preferentially affected the paclitaxel-residual cells (Figure 1B).

Among the six compounds that preferentially affected the paclitaxel-residual cells, the SMAC mimetics, Birinapant and BV-6 (Figure 1C), and the BCL-2 family inhibitors, ABT-263 and ABT-737 (Figure 1D), had the most potent inhibitory effects (> 100, 31.4, 10.4 and 3.13 fold reduction in the IC_50_, respectively). Although, the Smoothened/Hedgehog (SMO/HH) pathway antagonist BMS833923 (XL139) was also preferentially effective (∼3.19-fold) against paclitaxel-residual MDA-MB-231 cells (Figure 1E), its effect was cell-type specific, whereas the Ca+2-sensing receptor (CaSR) activator AMG-073 (Cinacalcet-HCl) (Figure 1E) was effective to a lesser extent (by 1.56 fold). The potency of these six compounds was further validated by at least three additional experiments. The high efficacy of ABT-263/737, as well as Birinapant and BV-6, which target different branches of the apoptotic machinery, strongly suggests that short-term paclitaxel treatment sensitizes residual MDA-MB-231 cells towards apoptotic targeted therapy. Similar effects have been reported following combined administration of taxanes, with the SMAC mimetics JP1400, Debio 1143 and Birinapant in non-small cell lung cancer (NSCLC) [14, 15] and breast cancer [16].

### Paclitaxel treatment sensitizes multiple TNBC cell lines to SMAC mimetics and BCL-2 family inhibitors

**Figure 2:**
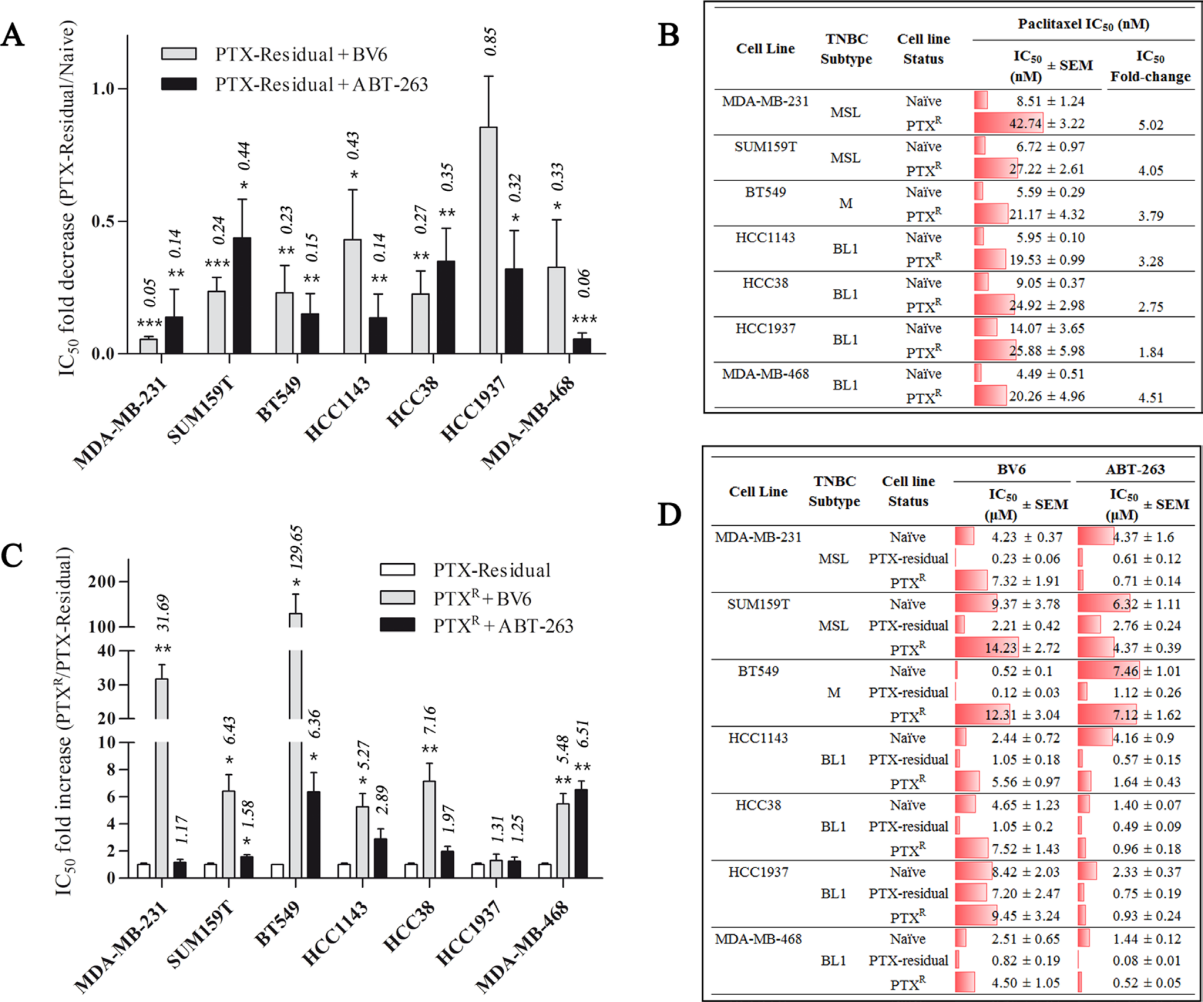
Effects of BV6 and ABT-263 on paclitaxel-residual and –resistant (PTX^R^) TNBC cell viability (**A**) Fold decrease of the IC_50_ for BV6 and ABT-263 in the indicated paclitaxel-residual TNBC cell lines compared to parental naïve cells. The results represent the values ± SEM from three independent experiments. (**B**) IC_50_ values of Paclitaxel for parental naïve and PTX^R^ TNBC cell lines that were generated following repeated cycles of drug pulse followed by recovery in drug-free medium. IC_50_ values are the average of three independent experiments, and where determined following 72 h of treatment. IC_50_ fold-change represents the ratio of IC_50_ values of PTX^R^ compared to naïve cells. (**C**) Fold-increase of the IC_50_ for BV6 and ABT-263 in PTX^R^ cells in the indicated 7 TNBC cell lines compared to paclitaxel-residual cells. The respective fold change is presented over each bar. The results represent the values ± SEM from three independent experiments. (**D**) IC_50_ values of BV6 and ABT-263 for parental naïve, paclitaxel-residual and PTX^R^ TNBC cell lines. PTX: Paclitaxel. PTX-residual: TNBC cells treated with paclitaxel IC_50_ for 96 h, followed by 96h recovery in drug-free medium. MSL: Mesenchymal stem cell-like; M: Mesenchymal; BL1: Basal-like 1. SEM: standard error of the mean.

The strong inhibitory effect of SMAC and BH3 mimetics on paclitaxel-residual MDA-MB-231 cells viability, led us to investigate whether short-term paclitaxel treatment could sensitize other TNBC cell lines to these apoptosis-inducing drugs (Supplementary Table 2). To this end, we determined the IC_50_ of ABT-263 and BV6 in six additional TNBC cell lines, including the basal-like (HCC1143, HCC38, HCC1937, MDA-MB-468) and the mesenchymal/mesenchymal stem-like (SUM159T, BT549) cell lines. Indeed, short-term treatment with paclitaxel sensitized the seven TNBC cell lines to both ABT-263 and BV6 regardless of their subtype or oncogenic mutations (Figure 2A). Paclitaxel-residual MDA-MB-231 cells were the most sensitive to BV6 (18.4-fold reduction in IC_50_) compared to the parental cells, whereas paclitaxel-residual MDA-MB-468 cells were particularly sensitive to ABT-263 (18-fold reduction in IC_50_). The remaining TNBC cell lines exhibited a 2.3-4.4-fold reduction in their IC_50_ for BV6 and 2.8-7.3 for ABT-263, with the exception of the *BRCA1*-mutant HCC1937 cells, which were marginally affected by BV6. Overall, these results suggest that both BH3 and SMAC mimetics could be effective therapeutic drugs following short-term treatment with paclitaxel.

### Paclitaxel-resistant cells induced by sustained paclitaxel treatment lose their sensitivity to SMAC mimetic but not to BCL-2 inhibitors

Given that chemotherapeutic agents often induce toxicity, chemotherapy treatments are frequently applied over-time by weekly, bi-weekly, or monthly cycles coupled with repeated recovery periods, in which drug resistance could be developed. We, therefore, hypothesized that acquired resistance over long-term exposure to repeated cycles of chemotherapy could elicit different responses to subsequent targeted therapy compared to the short-term treatment described in our HTS setup. To establish cellular models of long-term chemoresistance, we employed 4–6 repeated pulses of increasing concentrations of paclitaxel followed by recovery cycles, as previously described [17]. Using this protocol, we established seven paclitaxel-resistant (PTXR) TNBC cell lines, exhibiting approximately 2-5-fold increase in the IC50 of paclitaxel (Figure 2B).

We then examined the potency of SMAC and BH3 mimetics on these PTX^R^ TNBC cell lines. As depicted in Figure 2C, the PTX^R^ TNBC cell lines were de-sensitized to SMAC mimetics, but remained sensitive to ABT-263. Specifically, paclitaxel resistance was accompanied by a 5- to-7-fold increase in the IC_50_ of BV6 for SUM159, HCC38 and HCC1143 cells, and a 32-fold or even 130-fold increase in the case of MDA-MB-231 and BT549 cells, respectively (Figure 2C–2D). In contrast, ABT-263 was equally effective against PTX^R^ and paclitaxel-residual MDA-MB-231 cells (1.17 fold change in IC_50_). Although the remaining PTX^R^ cell lines were less responsive to ABT-263 than their paclitaxel-residual counterparts, they were still more sensitive (by 1.5-2.75 fold) compare to their corresponding parental paclitaxel-naïve cells, with the exception of BT549 cells (Figure 2D). These results suggest that targeting of different apoptotic pathways by two commonly used pro-apoptotic drugs can distinctly influence short- and long-term chemoresistance, and thus, could be effective at different time points after paclitaxel treatment.

### Short-term paclitaxel treatment affects gene expression of apoptotic-related genes including the TNFα pathway

**Figure 3:**
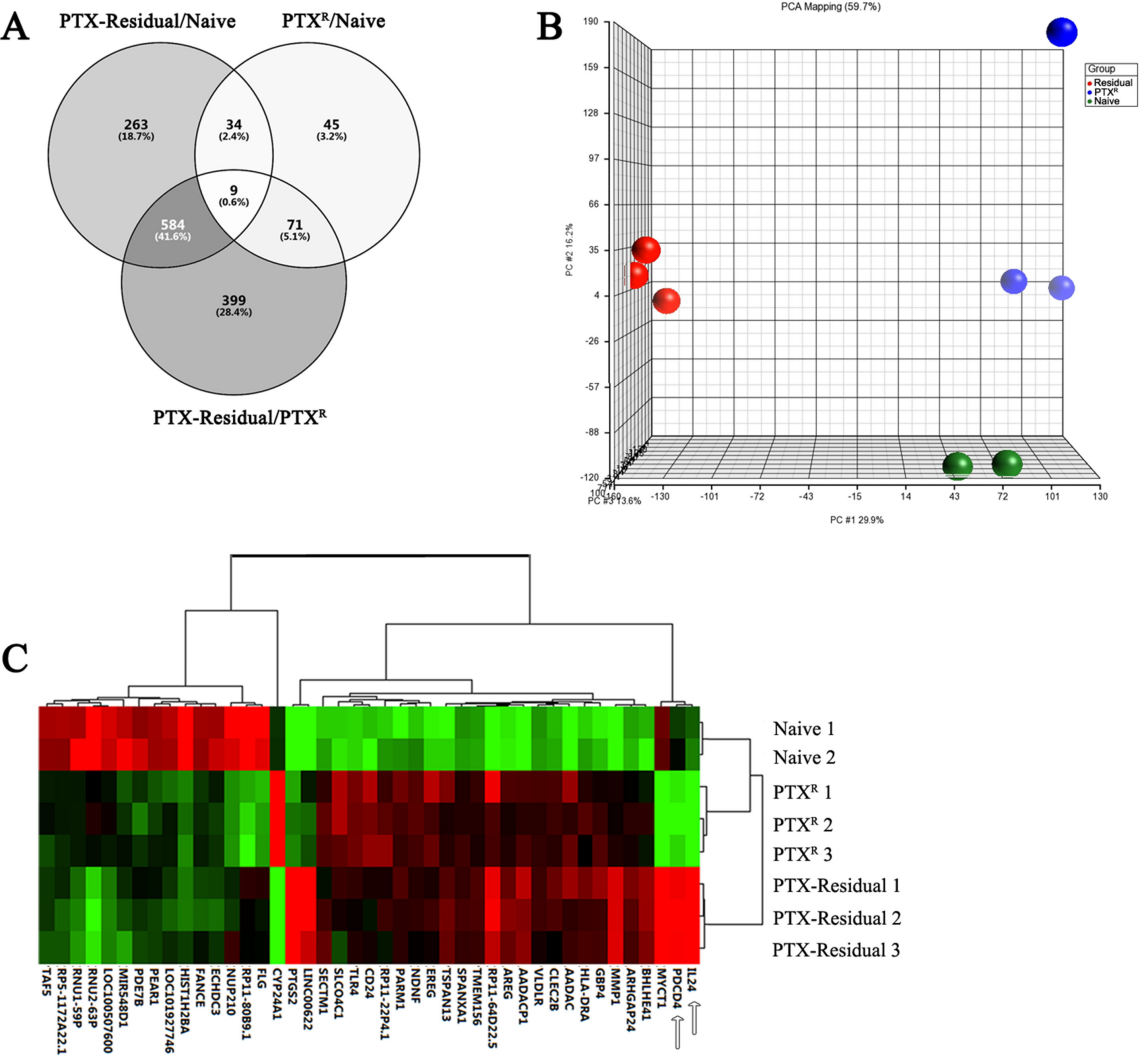
Affymetrix microarray expression profiling of parental naive, paclitaxel-residual and –resistant (PTX^R^) MDA-MB-231 cells (**A**) Venn diagram of common and differentially expressed genes (*p* < 0.05, fold change > 2 and < -2) between the three groups: PTX-residual/naïve, PTX^R^/naïve and PTX-residual/PTX^R^ cells. (**B**) Principal component analysis illustrating the variance between the different gene expression profiles of the biological replicates of naïve, PTX-residual and PTX^R^ cells. PTX-residual cells display a markedly different expression profile compared to the other two groups. (**C**) Hierarchical clustering of the 43 commonly affected genes (Venn diagram, panel A) between either paclitaxel-residual or –resistant cells versus the parental naïve controls (Java TreeView [48]).

The different sensitivity of paclitaxel-residual and resistant cells to SMAC and BH3 mimetics, led us to investigate the underlying mechanisms. To this end, a genome-wide gene expression profiling of parental naïve MDA-MB-231 cells, as well as paclitaxel-residual and PTX^R^ cells was assessed by Affymetrix Microarrays. The relevant datasets have been deposited in Gene Expression Omnibus (GEO) under the accession number GSE86839. ANOVA analysis of differential gene expression profiles indicated that 890 genes of the paclitaxel-residual cells were significantly altered (by ± 2 fold) compared to the naïve cells, while 1063 genes compared to the PTX^R^ cells (Figure 3A), and that 593 genes (43.6%) were commonly affected between the two groups (residual/naïve and residual/PTX^R^). In contrast, the gene expression profile of PTX^R^ cells was more related to the naïve cells and only 159 genes displayed significantly different levels, of which 43 were shared with the paclitaxel-residual cells (Figure 3A, 3C). Principal component analysis (PCA) showed that the gene expression profiles of paclitaxel-residual cells were markedly different from those of parental naïve and PTX^R^ cells (Figure 3B), which is consistent with previous studies employing single cell RNA-sequencing of MDA-MB-231 cells following paclitaxel treatment [18].

**Figure 4:**
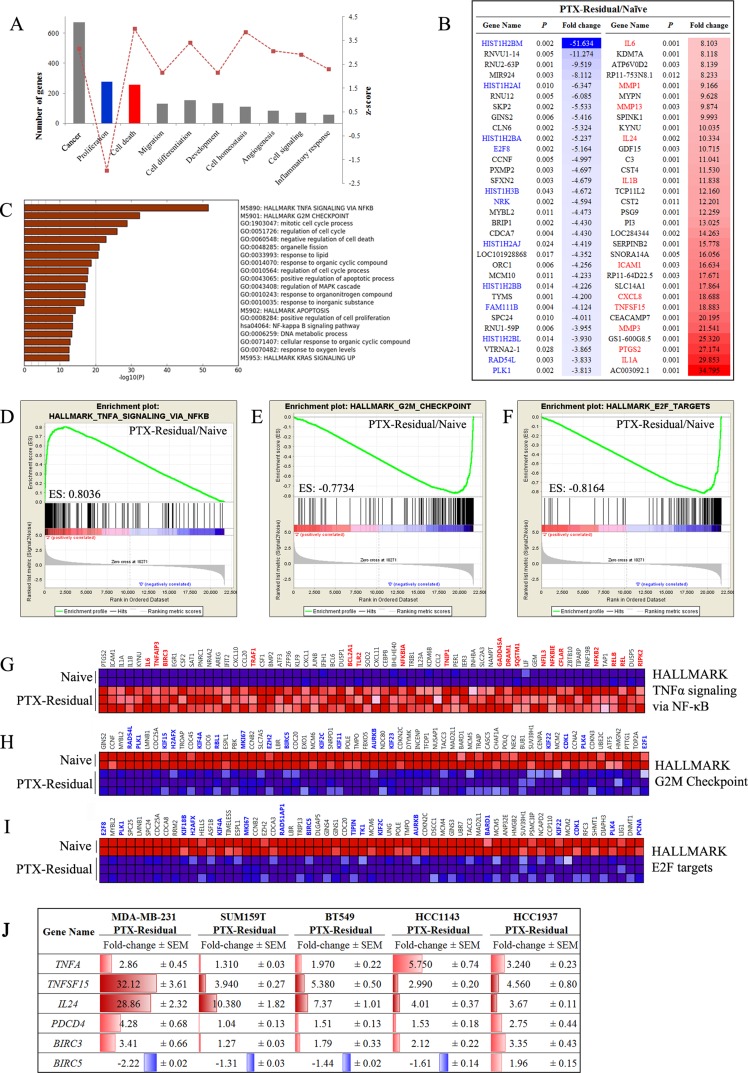
Gene expression analysis of paclitaxel-residual MDA-MB-231 cells (**A**) Most significantly affected biological processes according to Ingenuity Pathway Analysis in PTX-residual MDA-MB-231 compared to the parental naïve cells. (**B**) List of top 30 significantly down- and up-regulated genes in the paclitaxel-residual group (Partek Genomics Suite). The fold change values are given as linear ratios of the respective gene expression values. Significantly downregulated or upregulated genes implicated in cell cycle-related processes or cell-death/stress response are highlighted in blue or red, respectively. (**C**) Pathway enrichment analysis (Metascape server) of the IPA apoptosis-related genelist. The most enriched pathway is the Hallmark TNFα signaling via NFκB, whereas the cell cycle-related pathways are negatively enriched. (**D**–**F**) Enrichment plots (GSEA) of the Hallmark genesets related with TNFα/NFκB signaling (D), G2M mitotic checkpoint (E) and E2F targets (F). (**G**) Heatmap of the significantly enriched genes of the HALLMARK TNFa signaling via NFkB geneset (GSEA). Genes that are implicated in the response to SMAC or BH3 mimetics are highlighted with red. Heatmaps of the negatively enriched (**H**) Hallmark G2M Checkpoint geneset and (**I**) Hallmark E2F targets (GSEA). Important genes (e.g. *BIRC5*, *E2F1*, *AURKB*, *PLK1*, various kinesins) are highlighted with blue. (**J**) qRT-PCR analysis of the indicated genes in the indicated paclitaxel-residual TNBC cell lines and their corresponding parental cells was used to calculate the relative gene expression levels. The results are presented as fold change of expression in paclitaxel-residual cells compare to parental cells. The mean values ± SEM from three independent experiments are shown. Increase in gene expression is highlighted in red and decrease in blue.

Statistical assessment and pathway analysis of gene expression profiles of paclitaxel-residual cells indicated that the drug response was characterized by: (a) enrichment of TNFα/NFκB signaling, and upregulation of apoptosis-related genes (Figure 4A–4D, 4G, 4J), and (b) G2/M growth arrest (Figure 4A, 4C, 4E, 4H) regulated mainly by E2F transcription factors (Figure 4B, 4F, 4I). Functional enrichment analysis of differentially expressed genes in paclitaxel-residual cells, realized with QIAGEN's Ingenuity® Pathway Analysis software (www.qiagen.com/ingenuity), deduced a significant downregulation of cell-proliferation-related genes (*N* = 277), and an overall induction of genes implicated in cell death (*N* = 258) (Figure 4A). Moreover, pathway enrichment analysis of the cell death-related gene signature (Metascape server) showed that 21% of the genes were involved in the TNFα signaling pathway (Figure 4C), which markedly affects the cellular response to SMAC mimetics [19, 20]. Specifically, various TNF superfamily ligands were among the most upregulated genes in paclitaxel residual cells; *TNFSF10*, which encodes for TRAIL, displayed over 5-fold increase (Supplementary Figure 1) and *TNFSF15*, the gene for VEGI/TL1A, was the seventh most induced transcript (∼19-fold; Figure 4B, 4J). TNFSF15/TL1A, which signals via the DR3 and TR6/DcR3 [21], is a potent inducer of NF-κB, JNK, p38 MAPK and p42/p44 MAPK [22], and is implicated in cell cycle arrest and programmed cell death [23]. The TNFα/NF-κB downstream signaling cascade was also affected, as demonstrated by more than 2-fold upregulation of *NFKB2* and the NF-κB inhibitors *NFKBIA* and *NFKBIE* (Figure 4G), as well as over 4-fold downregulation of the *NRK* gene encoding for the NIK Related Kinase (Figure 4B).

These findings pinpoint the *a priori* sensitization of paclitaxel-residual cells to apoptosis-inducing agents, such as SMAC mimetics (Figure 1C), which function mainly via the extrinsic apoptotic pathway [19] and exhibit high efficacy in combination with autocrine TNFα signaling [20]. Importantly, upregulation of *TNFA* gene expression as well as *TNFSF15* was not confined to MDA-MB-231 cells, but was also observed in SUM159T, BT549, HCC1143 and HCC1937 cells in response to short-term paclitaxel treatment (Figure 4J). Likewise, *BIRC5* expression was downregulated in all the paclitaxel-residual lines except the *BRCA1*-mutant HCC1937 cells (Figure 4J). As *BIRC5* encodes the IAP family member protein survivin, which is implicated in resistance of breast cancer to apoptosis [24], its downregulation could sensitize paclitaxel-residual cells to pro-apoptotic drugs, like the SMAC mimetics. *BIRC3*, however, encoding the SMAC mimetics specific target cIAP2, was induced by short-term paclitaxel in all the cell lines (Figure 4J), and might prime the cells to SMAC mimetic targeting.

The paclitaxel-induced cell death-related signature was associated with induction of the GADD (Growth Arrest and DNA Damage) family members, *GADD45A* (2.2-fold) and *GADD45B* (1.4-fold) as well as the *DDIT3*/GADD153 (4.37-fold) transcription factor, and the well-characterized apoptosis inducers *IL24* and *PDCD4* (10.4- and 3-fold induction respectively; Figure 3C; Figure 4J) [25, 26]. *BCL2* was downregulated (2.3-fold) in the paclitaxel-residual cells, whereas *IL24* and *PDCD4* were among the 43 commonly affected genes between both the paclitaxel-residual or PTX^R^ and the parental cells (Figure 3C). Notably, induction of *IL24* expression was evident in all paclitaxel-residual TNBC cell lines, whereas *PDCD4* was upregulated in all cell lines except for SUM159T (Figure 4J), suggesting that short-term paclitaxel treatment induces upregulation of various apoptosis-inducing factors in TNBC, and therefore sensitizes the cells to pro-apoptotic drugs such as SMAC or BH3 mimetics.

### Gene expression profiling of paclitaxel-residual compared to paclitaxel-resistant cells

**Figure 5:**
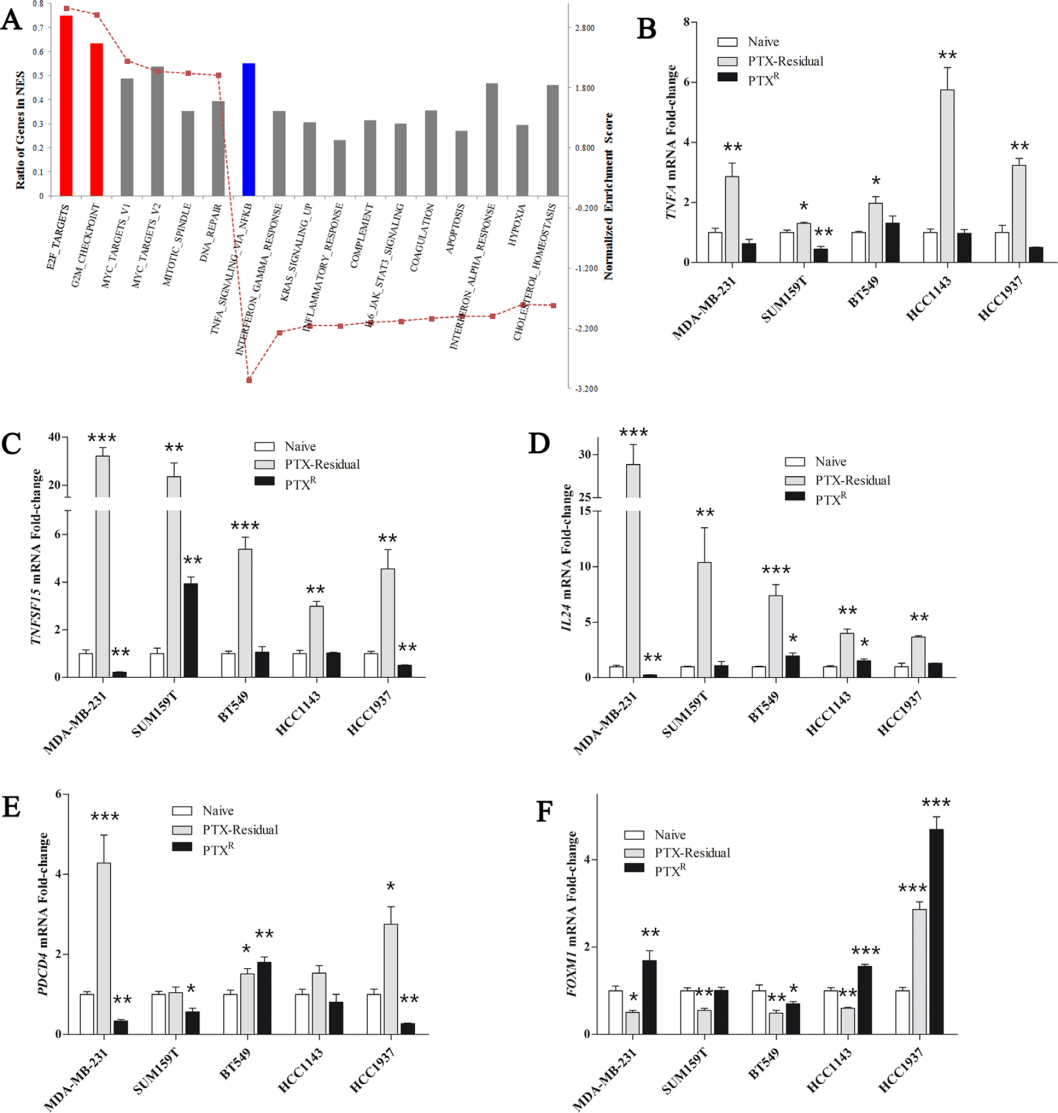
Transcriptional profile of paclitaxel-resistant cells compared to paclitaxel-residual cells (**A**) Geneset enrichment analysis (GSEA) for the paclitaxel-resistant (PTX^R^) dataset compared to the residual counterparts. The results show an inverse enrichment of the significantly affected genesets with negative enrichment of TNFα/NF-κB signaling (blue), and upregulation of cell cycle-related genes (red). (**B**–**F**) Relative expression of the indicated genes in PTX-residual and PTX^R^ TNBC cell lines. qRT-PCR analysis of the indicated genes in parental naïve, PTX-residual and PTX^R^ TNBC cells was used to calculate the fold changes between gene expression levels in PTX-residual or PTX^R^ cells and the respective naïve cells. The mean values ± SEM from three independent experiments are shown. The asterisks denote statistical significance *P < 0.05, **P < 0.01, ***P < 0.001. Statistical assessment was based on two-sided *t* test.

Pathway enrichment analysis of paclitaxel-residual cells suggests that short-term paclitaxel treatment sensitizes TNBC breast cancer cells to apoptosis mainly via upregulation of TNFα pathway coupled to downregulation of survivin transcription and G2/M cell cycle arrest mediated partially by the transcription factor E2F (Figure 4). Overall, the PTX^R^ MDA-MB-231 cells exhibited an inverse gene enrichment signature and were characterized by upregulation of the E2F regulatory network and negative enrichment of the TNFα/NF-κB signaling axis (Figure 5A), including decrease in *TNFA* gene expression, *TNFSF15, IL24* and *PDCD4* (Figure 5B–5E). Remarkably, the decrease in *TNFA, TNFSF15, IL24* and *PDCD4* expression was not confined to the PTX^R^ MDA-MB-231 cells, but was also observed in additional PTX^R^ TNBC lines (Figure 5B–5E). As shown in Figure 5B-E, despite the slight differences between the different TNBC lines, the upregulation of these four apoptosis-related genes in the paclitaxel-residual cells, and their concurrent downregulation or no change in the paclitaxel-resistant lines was obvious. In addition to these four genes, an upregulation of *FOXM1* transcription in PTX^R^ MDA-MB-231, HCC1143 and HCC1937 cells, concomitant with its downregulation in most of the paclitaxel-residual lines (MDA-MB-231, BT549, SUM159T and HCC1143) was observed (Figure 5F). The FOXM1 transcription factor is a proto-oncogene involved in cell cycle progression, cell proliferation, tumorigenesis and cancer progression [27], and many of its target genes (> 20) were downregulated in paclitaxel-residual MDA-MB-231 cells including *AURKB*, *CCNB1*, *PLK1*, *PLK2*, and the kinesin *KIF20A* (Figure 4B, 4H, 4I). These combined differences between short- and long-term paclitaxel treatments in TNFα pathway and G2/M cell cycle arrest mediated partially by the E2F and FOXM1 transcription factors (Figures 4, 5), could explain the susceptibility of short-term paclitaxel treatment to pro-apoptotic drugs (Figure 2A), and the desensitization of long-term paclitaxel treatment to SMAC mimetics (Figure 2C–2D), drugs that require the TNFR1-TNFα signaling pathway to induce apoptosis [15, 20].

Beside the above described genes, additional genes, including the Hypoxia-inducible factor 1-alpha *HIF1A* was differentially expressed in the paclitaxel-residual and -resistant cells (Supplementary Figure 2) and might reflect a hypoxia-related response during the recovery process from paclitaxel. The *HIF1A*-inducible gene *CA9*, which encodes carbonic anhydrase IX and regulates cellular pH to promote cancer cell survival [28] was induced in all PTX-residual cells, and its upregulation was sustained in MDA-MB-231, SUM159T, BT549 and HCC1937 PTX^R^ cells (Supplementary Figure 2). Other genes implicated in cellular detoxification, including *SLCO4C1 (*4-fold), *SOD3* (2.45-fold), and *ABCG2* (Supplementary Figure 1) were highly induced in PTX^R^ compare to PTX-residual MDA-MB-231 cells.

The ABC transporter ABCG2, which is involved in drug efflux and resistance, has recently been implicated in autophagy induction [29]. In accordance, PTX^R^ MDA-MB-231, SUM159T and HCC1143 cells, displayed increased levels of lipidated LC3 (Supplementary Figure 1). Increased autophagy was also evident during short-term paclitaxel treatment, as demonstrated by upregulation of the autophagy-associated genes *SQSTM1*, *DRAM1*, *RB1CC1* and *WIPI1* (Supplementary Figure 1) [30], possibly as part of the acute stress response to paclitaxel. Importantly, ABCG2 was found to be significantly upregulated in the non-basal TNBC TCGA subset (data not shown).

### Gene expression profile of *IL24*, *BIRC5* and *FOXM1* in breast cancer patients

**Figure 6:**
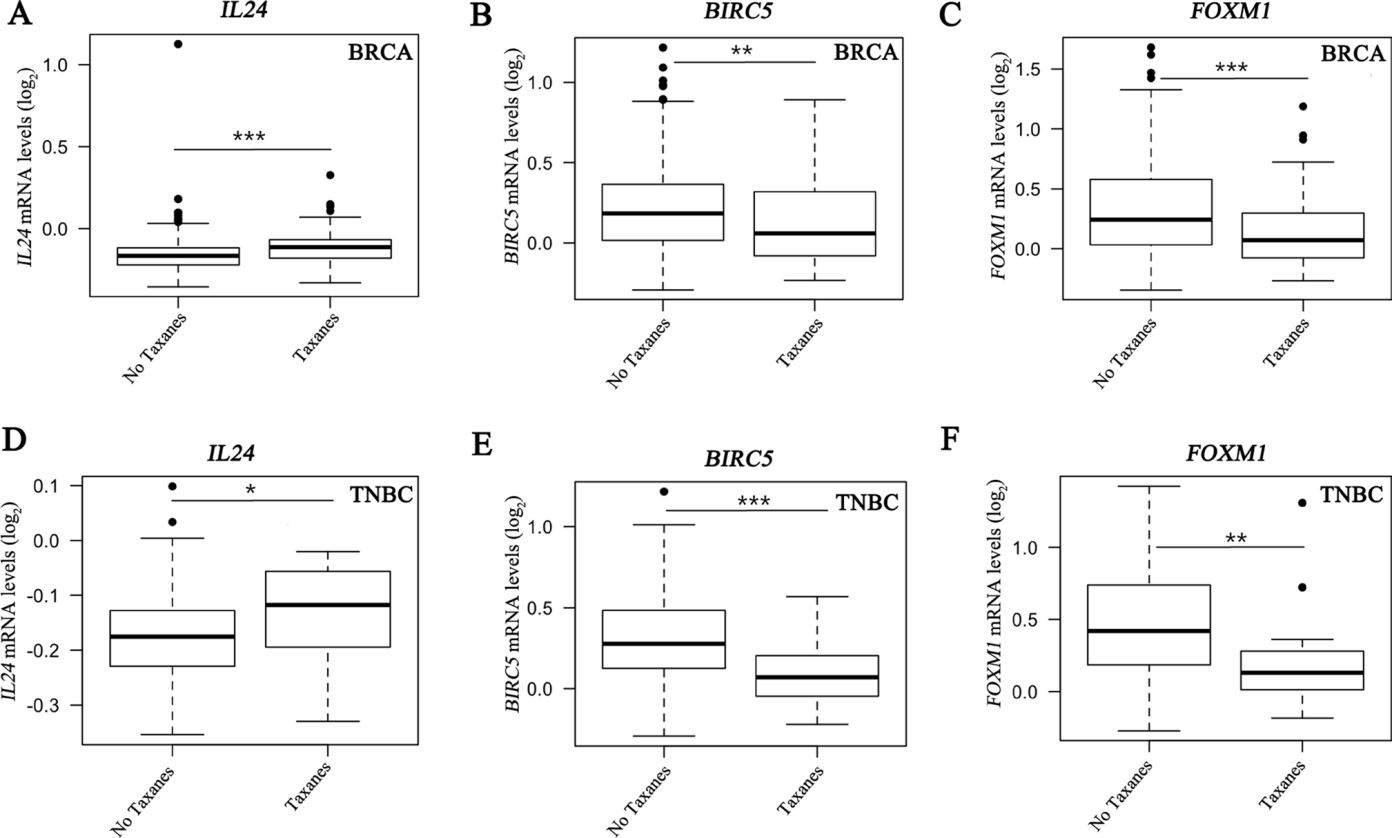
Effect of taxanes on *IL24, BIRC5* and *FOXM1* expression in breast cancer samples Gene expression levels of *IL24* (**A**, **D**), *BIRC5* (**B**, **E**) and *FOXM1* (**C**, **F**) in breast cancer patients (BRCA) irrespective of subtype (*N* = 488) and TNBC (*N* = 176) patients, from the publically available dataset GSE25066, treated with anthracyclines followed by sequential treatment (*N* = 185) or not (*N* = 303) with the taxanes taxol or taxotere. Differences in gene expression were assessed by a two-sided *t* test on Affymetrix U133A microarray data normalized using the SCAN method. The asterisks denote statistical significance *P < 0.05, **P < 0.01, ***P < 0.001.

We next assessed gene expression profile of 488 *HER2*-negative breast cancer patients of which 176 had TNBC disease using the GSE25066 dataset generated by MD Anderson Cancer Center (MDACC) [31]. The breast cancer patients were treated with taxanes (*N* = 185) following anthracycline-based regimens in a neoadjuvant setting [31, 32]. This analysis revealed a significant upregulation of *IL24* expression following taxane administration in clinical samples of both ER-positive and -negative breast cancer patients (Figure 6A), in particular in the ER-negative TNBC patients (Figure 6D). These findings are consistent with the upregulation of *IL24* expression in all the paclitaxel-residual TNBC cell lines (Figure 5D). Likewise, a statistically significant downregulation of *BIRC5* transcription as well as *FOXM1* expression was obtained in breast cancer patients irrespective of their subclass (Figure 6B, 6C), as well as in TNBC patients (Figure 6E, 6F). These results are in agreement with our *in vitro* analysis of *BIRC5* and *FOXM1* expression in paclitaxel-residual TNBC cells (Figures 4J, 5F), and further substantiate our findings. Collectively, these analyses suggest that a subset of paclitaxel-treated patients with increased *IL24*, and reduced *BIRC5* and *FOXM1* expression levels could benefit from SMAC or BH3 mimetics treatment.

### Secreted-factors from paclitaxel-residual cells sensitize cells to SMAC mimetics

**Figure 7:**
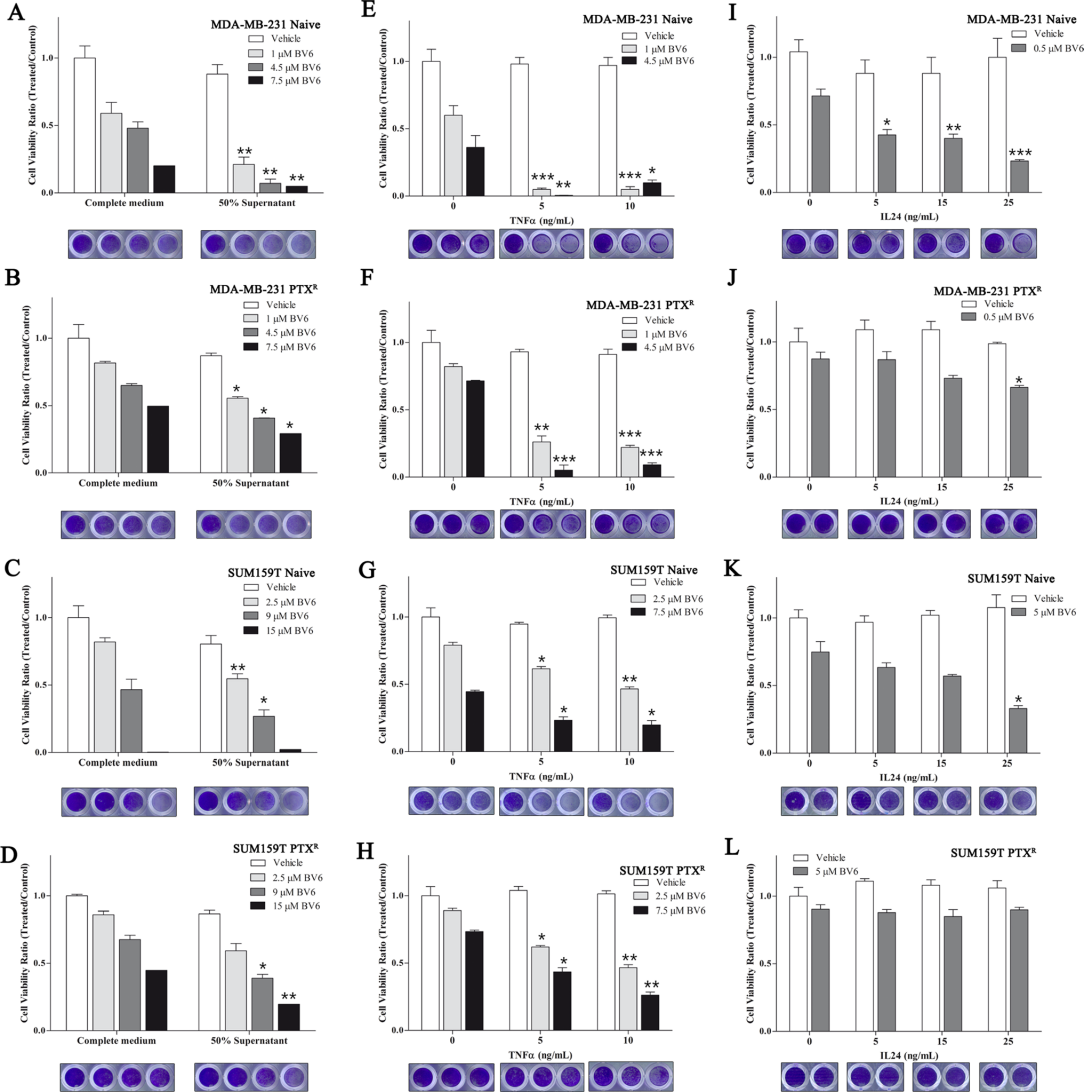
Secreted factor(s) by paclitaxel-residual cells sensitize cells to BV6 (**A**–**D**) Supernatants of paclitaxel (PTX)-residual cells sensitize cells to BV6. Parental naïve or PTX^R^ MDA-MB-231 (A, B) and SUM159T (C, D) cells were incubated with supernatants (50% in complete medium) of the corresponding paclitaxel-residual cells or control untreated cells (control medium), either alone or in combination with different concentrations of BV6 as indicated. Cell viability was measured after 72 hr using the Celltiter Blue assay, and the ratio between viability of cells grown in the presence of BV6 in control or PTX-residual derived media to those grown in the absence of BV6 was calculated. (**E**–**H**) TNFα sensitizes cells to BV6. Parental naïve or PTX^R^ MDA-MB-231 (E, F) and SUM159T (G, H) were incubated with the indicated concentrations of TNFα in the presence or absence of BV6 for 72 hr. Cell viability was measured as described above, and the ratios between cell viability in the presence of both BV6 and TNFα to the viability of cells in the presence of TNFα alone at the indicated concentrations are shown. (**I**–**L**) IL24 sensitizes cells to BV6. Parental naïve or PTX^R^ MDA-MB-231 (I, J) and SUM159T (K, L) were incubated with the indicated concentrations of IL24 in the presence or absence of BV6 for 72 hr. Cell viability was measured as described above, and the ratio between cell viability in the presence of both BV6 and IL24 to the viability of cells in the presence of IL24 alone at the indicated concentration was calculated. Statistical assessment of significant differences in cell viability between cells that were exposed to the combined treatment (BV6 and supernatant/TNFα/IL24) compared to cells that were treated with BV6 alone was based on two-sided *t* test. The results represent the mean values ± SEM of three independent experiments. The asterisks denote statistical significance *P < 0.05, **P < 0.01, ***P < 0.001. Crystal violet staining below each panel demonstrates the differences in cell viability in each condition.

As TNFSF15 and IL24 are secreted factors implicated in apoptotic pathways [33, 34], which were upregulated in the paclitaxel-residual cells (Figure 4B, 4J; Figure 5C, 5D), they might sensitize paclitaxel-residual cells to BV6 via an autocrine loop, and thus, could also sensitize naïve or PTX^R^ cells to this inhibitor. To explore this possibility, we incubated naïve and PTX^R^ MDA-MB-231 and SUM159T cells with supernatants of their paclitaxel-residual counterparts and examined their response to BV6. As shown in Figure 7A–7D, supernatants of paclitaxel-residual cells slightly reduced the viability of parental and PTX^R^ cells, but substantially potentiated the effect of BV6 on parental MDA-MB-231 (Figure 7A) and to a lesser extent, yet significant, on parental SUM159T (Figure 7C) or PTX^R^ MDA-MB-231 (Figure 7B) and SUM159T (Figure 7D) cells. Specifically, treatment of MDA-MB-231 naïve cells with 1 μM BV6 in the presence of supernatants from paclitaxel-residual cells reduced cell viability by more than 50% compared to treatment with BV6 alone (Figure 7A), suggesting that factor(s) secreted from paclitaxel-residual cells could sensitize the cells to BV6. However, the effects were more profound in MDA-MB-231 cells compared to SUM159, suggesting that secreted factor(s) released from paclitaxel-residual cells mediate, at least partially, the increased sensitivity to BV6 in a cell-type specific manner. To examine whether these effects are mediated by TNFα or IL24, we exposed naïve and PTX^R^ MDA-MB-231 and SUM159T cells to increasing concentrations of TNFα (Figure 7E–7H) or IL24 (Figure 7I–7L), in the presence or absence of BV6. As seen in Figure 7E–7H, TNFα substantially enhanced the effect of BV6 on both naïve and PTX^R^ MDA-MB-231 (Figure 7E, 7F) and SUM159T (Figure 7G, 7H) cells. However, IL24 could sensitize the parental MDA-MB-231 (Figure 7I) and SUM159 (Figure 7K) cells to BV6, but had no significant effect on the PTX^R^ cells (Figure 7J, 7L). These results suggest that TNFα and IL24 mediate, at least in part, the cellular sensitivity to SMAC mimetics following short-term paclitaxel treatment.

## DISCUSSION

In the present study, we demonstrated that targeting of different apoptotic pathways could distinctly affect the outcome of short- and long-term paclitaxel-treatment (Figures 1, 2) and overcome chemoresistance. By using a HTS setup with 320 small molecule inhibitors, we identified the SMAC (Birinapant, BV6) and the BH3 (ABT-737, ABT-263) mimetics as drugs that can preferentially and effectively eliminate MDA-MB-231 cells that escaped short-term paclitaxel treatment (Figure 1). Similar results were obtained for 6 additional TNBC (HCC1143, HCC38, HCC1937, MDA-MB-468, SUM159T, BT549) cell lines, irrespective of their molecular subtype or driver mutations (Figure 2). These findings suggest that targeting of IAPs or BCL-2 family members could be beneficial for paclitaxel-treated TNBC patients. Indeed, it was shown that SMAC mimetics potentiate paclitaxel-mediated ovarian cancer cell death *in vitro* and *in vivo* [35] and similar effects were reported for NSCLC [14, 15] and breast cancer [16]. Likewise, administration of navitoclax (ABT-263) enhanced taxane-based treatment of ovarian cancer [36] and NSCLC [37].

Nevertheless, persistent paclitaxel treatment over 6–8 months results in paclitaxel-resistant cells that lost sensitivity to SMAC mimetics but not to BCL-2 inhibitors (Figure 2). Interestingly, Kutuk et *al*. reported that simultaneous administration of ABT-737 with paclitaxel can also sensitize paclitaxel-resistant MCF7 cells to taxol [38], implying that TNBC as well as ER-positive patients could be benefit from this drug combination.

Our analysis showed that short-term challenge with paclitaxel induces transcriptional signatures characterized by cell-cycle arrest, possibly mediated by E2F and FOXM1 transcription factors, and by TNFα/NF-κB signaling enrichment, as well as induction of an apoptotic phenotype, including downregulation of the FOXM1 and E2F target gene survivin (Figures 4, 5). Notably, *BIRC5* and *FOXM1* expression was also reduced in taxane-treated breast cancer patients (Figure 6), and *FOXM1* expression was upregulated in most of the PTX^R^ TNBC cell lines (Figure 5F) consistent with its established role in drug resistance to genotoxic agents, such as taxane and epirubicin [27]. Importantly, de Moraes et *al* [39] have reported that FOXM1 protein expression is significantly associated with survivin and XIAP levels in patients with IIIa stage breast invasive ductal carcinoma. In addition, simultaneous expression of FOXM1, survivin, and nuclear XIAP in these patients was associated with significantly worst overall survival. Other pro-apototic genes including *IL24* and *PDCD4* were upregulated by short-term paclitaxel treatment (Figure 4J) and *IL24* upregulation was also evident in taxane-treated TNBC patients (Figure 6B). These results could explain the sensitivity of paclitaxel-residual cells to SMAC or BH3 mimetics (Figure 2A).

SMAC mimetics have been generally well-tolerated in clinical trials [19]. They target the IAP family (cIAPs, XIAP, ML-IAP) and consequently increase their E3 ligase activity, autoubiquitination and degradation via the proteasome [19]. This leads to NIK accumulation, non-canonical NF-*κ*B activation and upregulation of NF-*κ*B target genes, such as *TNFA* [20] and induction of apoptosis via a RIP1/FADD/caspase-8 cytosolic complex in an autocrine manner [19]. Despite the overall good properties of the SMAC mimetics, these inhibitors require the presence of autocrine TNF*α* signaling for optimal activity [20]. Indeed, we found that short-term paclitaxel treatment induced upregulation of *TNFA* expression in all the TNBC lines that were examined (Figure 4J), and that negative enrichment of TNFα signaling pathway was observed during development of adaptive paclitaxel resistance concomitant with desensitization to SMAC mimetics (Figures 2C–2D, 5A). Moreover, TNFα could partially sensitize the naïve and/or the paclitaxel-resistant TNBC cells to BV6 (Figure 7E–7H). We also observed a partial effect of IL24 (Figure 7I–7L), but neither of them could exclusively reconstitute the sensitivity of the residual cells, implying that other secreted factors might be involved. Potential candidates could be other TNF superfamily ligands namely TRAIL and VEGI/TLA1 encoded by the *TNFSF10* and *TNFSF15* genes respectively, which were highly upregulated in paclitaxel-residual MDA-MB-231 cells (5.2- and 18.9-fold respectively; Figure 3) but not in the PTX^R^ cells. Both cytokines participate in apoptotic processes; TRAIL is a major component of the extrinsic apoptotic pathway, which induces apoptosis in cancer cells, albeit with disappointing results in clinical trials [40], while VEGI/TLA1, the only known ligand of Death Receptor 3, can inhibit the proliferation of breast carcinoma and increase breast cancer patient survival [22].

In contrast to SMAC mimetics, the BCL-2 family inhibitor, ABT-263 maintained its potency following long-term treatment with paclitaxel (Figure 2B) suggesting that targeting of BCL-2 family members could be a better therapeutic strategy in combination with taxol following a long recovery period. However, ABT-263, which inhibits BCL-2, BCL-xL and BCL-W, has considerable significant side-effects and can induce febrile neutropenia due to BCL-xL inhibition, even when applied as a single agent or simultaneously with docetaxel in clinically effective concentrations [41]. Hence, BCL-2 selective inhibition might be a safer alternative, but its potency could be limited. Indeed, the BCL-2 selective inhibitor, ABT-199 was not effective against paclitaxel-residual TNBC cells in our HTS (Figure 1, Supplementary Table 1). Nevertheless, we found that the basal-like TNBC lines are more sensitive to ABT-263 compare to the MS/MSL lines (Figure 2D), implying that ABT-263 could be more suitable for paclitaxel-treated basal-like TNBC patients.

So far, the efficacy of the combination of taxanes with SMAC or BH3 mimetics has been evaluated following simultaneous administration, which might be clinically irrelevant due to their high toxicity [3, 41, 42]. Pre-treatment with paclitaxel, as described in our study, sensitized the cells to SMAC and BH3 mimetics (Figure 1) and concurrently triggered reprogramming in gene expression profiles (Figure 3A), consistent with previous studies involved in sequential drug administration. For example, the EGFR inhibitor erlotinib sensitized EGFR-overexpressing TNBC cells to doxorubicin [43], whereas low dose metformin or the TopoI inhibitor SN38 increased the DNA damage response in ovarian cancer cells [44]. In both studies, the compounds increased apoptotic cell death, which rendered the cancer cells more vulnerable to the cytotoxic effect of chemotherapy.

Herein we propose an inverse scheme, where paclitaxel is administered as first agent, mimicking the neoadjuvant setting commonly implemented for the clinical management of TNBC with chemotherapy being the first line approach [3]. This strategy might be relevant for TNBC patients that did not respond well to a paclitaxel-containing regimen and thus sensitize the residual cancer cells to the subsequent addition of apoptosis-inducing compounds.

Taken together, in this study we identified potent therapeutic drugs that preferentially and effectively target paclitaxel-residual disease, and could thus minimize the chemotherapy-associated side effects. We further proposed alternative therapeutic strategies for short- and long-term residual disease, which could be of beneficial for different sub-populations of paclitaxel-treated TNBC patients.

## MATERIALS AND METHODS

### Breast cancer cell lines

TNBC cell lines were obtained from ATCC (Manassas, VA, USA), and were maintained in low passage. MDA-MB-231 cells were grown in DMEM (Gibco BRL, Grand Island, NY, USA). SUM159T were grown in 1:1 DMEM:F12 medium supplemented with 5% FBS, 10 μg/ml insulin and 0.5 μg ml-1 hydrocortisone. MDA-MB-468, HCC-1143, HCC-38, HCC-1937, and BT549 cell lines were cultured in RPMI 1640 (Gibco BRL) medium, further supplemented with 0.023 IU ml-1 insulin for BT549 cells. Unless otherwise stated, growth media were supplemented with 10% fetal bovine serum (FBS), 2 mm Glutamine, and penicillin/streptomycin. Cells were cultured at 37°C in a humidified incubator in the presence of 5% CO_2_.

### Reagents and antibodies

Paclitaxel was obtained from Calbiochem (Merck Millipore, Billerica, MA, USA). ABT-263 and BV6 for *in vitro* use were purchased by APEX Bio (Houston, TX, USA). The custom-made library of 320 small molecule compounds was purchased by Selleck Chemicals (Houston, TX, USA). Rabbit polyclonal anti-human LC3 (1:1,000) was kindly provided by Prof. Z. Elazar (Weizmann Institute of Science). Mouse monoclonal p62/SQSTM1 antibody (clone 2C11; 1:1,000) was purchased from Novus Biologicals (Littleton, CO, USA). Mouse monoclonal anti-human ATG5 (clone ATG5-18; 1:3,000) was purchased from Sigma. Mouse monoclonal anti-human GAPDH antibody (clone 6C5; 1:1,000) was obtained from EMD Millipore (Darmstadt, Germany).

### Development of paclitaxel-resistant breast cancer cell lines

Paclitaxel-resistant cell lines were developed by sequential cycles of drug pulse followed by recovery in drug-free medium, as previously described [17]. In brief, MDA-MB-231, SUM159T, BT549, HCC1143, HCC38, HCC1937, MDA-MB-468 cells were initially treated with IC_50_ concentrations of paclitaxel (Figure 2B) for 96 h. Subsequently, the cells were left to recover in drug-free media until they reached normal proliferation rates before the next cycle of treatment. Following up to 6 cycles of paclitaxel treatment, depending on the cell line, paclitaxel-resistant cell lines were cultured in complete culture media, in the absence of paclitaxel.

### High-throughput small molecule compound screen

For the identification of small molecule compounds that specifically target paclitaxel-residual cells, 4 × 10^3^ paclitaxel-naïve cells MDA-MB-231 cells were seeded in white flat bottom 384-well microplates (Greiner Bio-One, Frickenhausen, Germany) with a 384-multidrop dispenser (Thermoscientific, Waltham, MA, USA), left to proliferate for 24 h and were subsequently treated with 8.5 nM paclitaxel for 96 h. Following recovery in drug-free media for 96 h, paclitaxel-residual cells were incubated for 72 h with a library of 320 small molecule compounds. The compounds were plated in 5 different concentrations in 5-fold dilutions covering a 625-fold concentration range, centered around the reported EC_50_ of the drugs where available, using the Echo 550 Liquid Handler (Labcyte, Sunnyvale, CA, USA). The assay included as controls: (a) cells treated with paclitaxel for 96 h, recovered for 96h in drug-free medium, and subsequently treated with dimethyl sulfoxide (DMSO) for 72 h, and (b) cells treated with the compounds for 72 h, henceforth referred to as “paclitaxel-naïve”. The first control group served as a cut-off point for paclitaxel-induced cell death at the endpoint; the second group was implemented in order to assess the effect of paclitaxel to the efficacy of the compounds of interest. Paclitaxel-naïve cells were seeded at a density of 1.5 × 10^3^ cell/well 24 h prior to the addition of the compounds. The HTS was realized in parallel on paclitaxel-residual and control cells. Cell viability was assessed at the endpoint utilizing the CellTiter Glo luminescent cell viability assay, according to manufacturer's instructions (Promega, Madison, WI, USA). Luminescence was measured using a PHERAStar Plus plate reader (BMG Labtech, Ortenberg, Germany). The automated HTS workflow was carried out at the established infrastructure of the Israeli National Center of Personalized Medicine of the Weizmann Institute of Science. Data analysis was realized utilizing the GeneData Screener (Genedata, Basel, Switzerland) and Graph Pad Prism (GraphPad Software Inc, La Jolla, CA, USA) software.

### Drug sensitivity testing

For the determination of IC_50_ values for paclitaxel, and the manual validation of HTS results, cell viability was assessed using the Celltiter Blue fluorescent assay (Promega). Cells were seeded 24h prior treatment in black 96-well plates, at concentrations between 0.5–1.6 × 10^4^ cells/well, depending on the cell line. Viability was assessed 72 h following the addition of the compounds of interest following incubation with Celltiter Blue reagent for up to 2 h, depending on the cell line, at 37°C. The fluorescent signal was measured at 560_Exc_/590_Em_ nm with a TECAN fluorescent reader (Mannedorf, Switzerland). Changes in cell viability are presented as the ratio of viable cells between treated and the respective Mock-treated control cells. The results were verified by crystal violet staining.

### Western blotting

Preparation of cell lysates, SDS-PAGE, and western blotting (WB) were realized as previously described [45].

### Preparation of supernatants

Supernatants from paclitaxel-residual cells were collected at the endpoint of 96h paclitaxel treatment/96 h recovery in drug-free medium. Supernatants were centrifuged for 15 min at 1,000 *xg* in order to remove cell debris and were immediately transferred to the target cells, at a concentration of 50% with the addition of complete medium. In all instances, cells treated only with supernatants were used as negative control. Cell viability was measured following 72h.

### RNA extraction and qRT–PCR

Total RNA was extracted utilizing the TRI Reagent (Sigma). For cDNA preparation, 2 μg of RNA, extracted with the TRI Reagent (Sigma), were reverse transcribed using oligo(dT) primer and M-MLV reverse transcriptase (Promega). Quantitative real-time PCR analysis was performed using SYBR Green I, according to the manufacturer's guidelines using the ABI StepOnePlus 7500 Real-time PCR system (Applied Biosystems; Thermoscientific). All experiments were carried out in triplicates and the levels of mRNA were normalised to GAPDH mRNA. Unless otherwise stated, real-time PCR primers were designed using the Primer-BLAST designing tool. Relative mRNA levels were calculated using the ΔΔC_T_ method. The oligonucleotide primers used in the study are listed in Supplementary Table 3.

### Gene expression microarray

Total RNA for microarray analysis was extracted using the TRI Reagent. RNA purity and integrity were checked on Agilent Bioanalyzer 2100. cDNA synthesis and amplification for microarray analysis was performed according to Affymetrix (Santa Clara, CA, USA) protocols at the Weizmann Institute of Science Microarray core facility. Whole genomic expression profile of three biological replicates per sample was obtained using Affymetrix HumanGenome 2.0 ST arrays. Differential gene expression detected by gene expression microarrays was validated by qRT-PCR where necessary. The relevant datasets have been submitted in GEO under the accession number GSE86839.

### Microarray data analysis

Following pre-processing and quality control, raw Affymetrix microarray probe-level data were normalized using the GC Robust Multi-array Average (GCRMA) background adjustment, and were log_2_ transformed using Partek Genomics Suite prior to analysis. ANOVA-based statistical assessment of differentially expressed genes between experimental conditions was conducted with Partek software following exclusion of genes with low expression. Benjamini & Hochberg multiple test correction was employed to reduce the number of false positive results.

Changes in gene expression profiles between naïve, paclitaxel-residual and paclitaxel-resistant MDA-MB-231 cells were assessed utilizing the following strategies. In order to identify differentially expressed genes that are relevant for the increased efficacy of BV6 and ABT-263 against paclitaxel-residual cells, the gene expression pools of paclitaxel-residual MDA-MB-231 cells were compared against the profiles of naïve parental cells. The identification of genes that might be implicated in the observed resistance to BV6 and the preservation of ABT-263 toxicity in the paclitaxel-resistant cells was realized by comparing the gene expression levels of (a) paclitaxel-residual/paclitaxel-resistant and (b) paclitaxel-resistant/naïve expression datasets.

Functional analysis, and upstream regulator analysis was realized with the QIAGEN's Ingenuity^®^ Pathway Analysis (IPA^®^, QIAGEN Redwood City, www.qiagen.com/ingenuity) software and was based on genes with adjusted *P* values less than 5% and fold change > 2 and < -2. Gene set enrichment analysis was conducted utilizing the desktop application of GSEA package (Broad Institute, MIT) [46], employing the MSigDB 5.1 genesets, with special emphasis on the Hallmark [47], canonical and GO genesets. Expression heat maps of differentially expressed genes were built using the GSEA platform. Cluster analysis of samples was performed using Pearson's correction and complete linkage cluster algorithm using Java TreeView [48].

### Analysis of publically available datasets

Retrospective statistical assessment of gene expression within clinicopathological parameters and patients’ survival was based on the level 3 RNA-seq TCGA gene expression data for breast cancer (BRCA). Gene expression alterations following taxane treatment and possible correlation with pCR or invasive residual disease (RD) was assessed utilizing previously published microarray datasets, namely GSE25066 [31], GSE41998 [49], GSE22358 [50], GSE22226 [51], and GSE32646 [52]. TNBC subtyping was realized according to Lehmann et al [2, 32].

The Affymetrix U133a microarrays from GSE25066 were normalized using the single-sample microarray normalization (SCAN) method [53] and annotated using a custom definition file from Brainarray (version 20) mapping the array probes to Entrez gene IDs [54]. Differences in expression of individual genes were evaluated from a two-sided *t*-test. To calculate the pathway enrichment between pCR and RD patients, a Generally Applicable Gene-set Enrichment (GAGE) was applied [55] using the Hallmark Gene sets from the MSigDB version 5.1.

## SUPPLEMENTARY MATERIALS FIGURES AND TABLES




