# Supplementary material for: Targeting of apoptotic pathways by SMAC or BH3 mimetics distinctly sensitizes paclitaxel-resistant triple negative breast cancer cells
Source: Oncotarget. 2017 Feb 6;8(28):45088–104. doi: 10.18632/oncotarget.15125 (PMC5542169; doi:10.18632/oncotarget.15125)
Supplement: Supplementary file 1 [file oncotarget-08-45088-s001.pdf]

Targeting of apoptotic pathways by SMAC or BH3 mimetics distinctly sensitizes paclitaxel-resistant triple negative breast cancer cells

Supplementary Materials

A

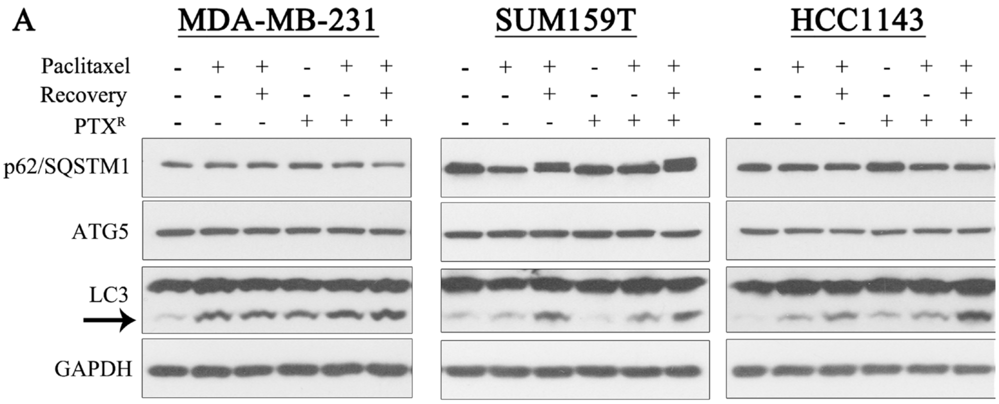

B

Gene Name	PTX-Residual/Naive		PTX^R/Naive	
	P	Fold-change	*P*	Fold-change
TLR4	0.001	2.525	0.003	3.484
WIPI1	0.002	2.041	0.081	1.205
IL24	0.003	10.361	0.038	-3.365
DRAM1	0.009	2.184	0.957	NS
SQSTM1	0.010	2.159	0.495	NS
RB1CC1	0.015	2.025	0.976	NS
ABCG2	0.071	1.433	0.015	3.203
TNFSF10	0.003	5.183	0.943	1.023

C

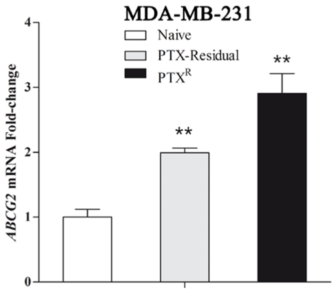

Supplementary Figure 1: Paclitaxel treatment induces autophagy-associated markers in TNBC cells. (**A**) Protein expression levels of the autophagy-related proteins p62/SQSTM1, ATG5, and LC3 in parental and paclitaxel-resistant (PTX^R) MDA-MB-231, SUM159T and HCC1143 TNBC cells. Protein levels were monitored in (a) Mock-treated, (b) cells treated with paclitaxel for 96 h and (c) paclitaxel-residual cells (96 h paclitaxel treatment followed by 96 h recovery). The arrow indicates the lipidated form of LC3. GAPDH was used as a loading control. (**B**) Differential expression of autophagy-related genes in paclitaxel-residual and PTX^R MDA-MB-231 cells by Affymetrix HuGene 2.0 ST microarray. (**C**) qRT-PCR analysis of *ABCG2* in paclitaxel-residual and -resistant (PTX^R) MDA-MB-231 cells. Relative gene expression levels were calculated compared to the corresponding naïve parental cells. The results are presented as fold change of expression in PTX-residual and PTX^R cells compare to naïve parental cells. The mean values ± SEM from three independent experiments are shown. The asterisks denote statistical significance *$P < 0.05$, **$P < 0.01$, ***$P < 0.001$. P: *P* value. NS: non-significant.

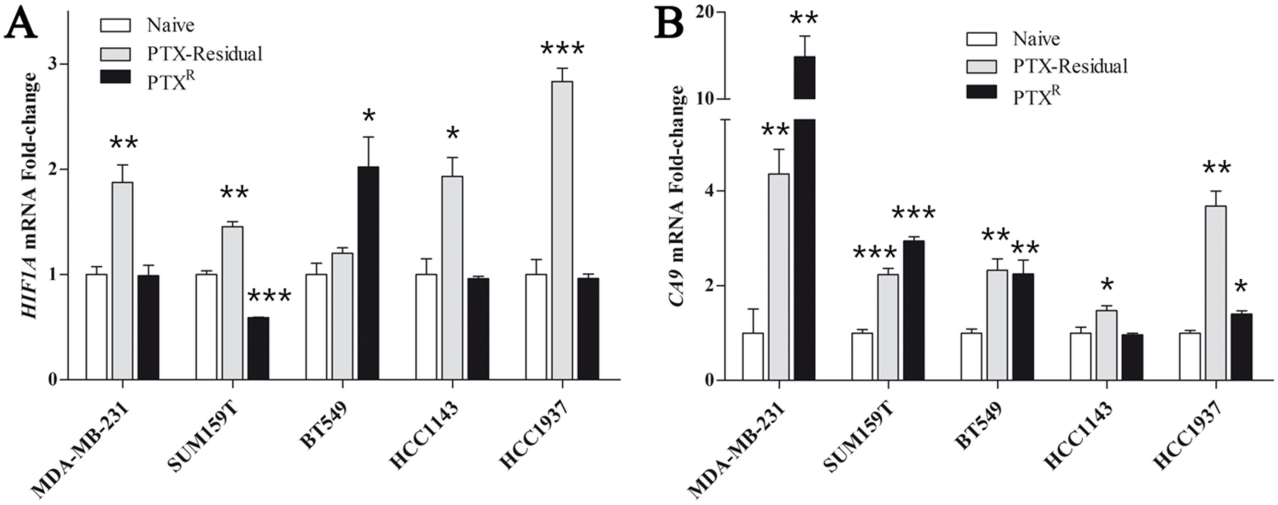

Supplementary Figure 2: Expression levels of *HIF1A* and *CA9* in paclitaxel-residual and -resistant (PTX^R) TNBC cells. qRT-PCR analysis of *HIF1A* (**A**) and *CA9* (**B**) in the indicated paclitaxel-residual and PTX^R TNBC cell lines. Relative gene expression levels were calculated compared to the corresponding naïve parental cells. The results are presented as fold change of expression in PTX-residual and PTX^R cells compare to naïve parental cells. The mean values ± SEM from three independent experiments are shown. The asterisks denote statistical significance *$P < 0.05$, **$P < 0.01$, ***$P < 0.001$.

Supplementary Table 1: Complete list of the 320 small molecule compounds employed in the High-Throughput Screen. See_Supplementary_Table 1

Supplementary Table 2: Mutation and expression profile of TNBC cell lines that were included in the present study

Cell line	Subtype[a]	Mutation profile	Expression profile
MDA-MB-231	MSL	*BRAF*, *CDKN2A, KRAS*, *NF2*, *TP53*, *PDGFRA*	PTEN+wt, CDKN2-null, p53+ve
SUM159T	MSL	*C-MYC amplification, PIK3CA, TP53, HRAS*	EGFR+ve, ERBB-2+ve, PTEN-wt
BT549	M	*PTEN, RB1, TP53, GAB1, MMP1*	p53+ve, PTEN-null
HCC1143	BL1	*TP53*, *TP63*, *MEK1*, *MAP2K6*, *MAP3K14*, *NCAM1*, *SMAD2*, *TGFB2*, *MMP1*	p53+ve, PTEN-wt
HCC38	BL1	*PTEN, CDKN2, TP53*	p53+ve, PTEN-null
HCC1937	BL1	*BRCA1, PTEN*, *TP53, MAPK13, MDC1*	p53−ve, PTEN-null
MDA-MB-468	BL1	*PTEN, RB1, SMAD4, TP53*	PTEN-null, EGFR overexpression

[a]*MSL: Mesenchymal stem cell-like, M: Mesenchymal, BL1: Basal-like 1.*

Supplementary Table 3: Oligonucleotide primers used for qRT-PCR

Primer	Sequence (5′-3′)	Source
ABCG2 F	GGA TTT ACG GCT TTG CAG CAT	This study
ABCG2 R	TCT TCG CCA GTA CAT GTT GCA T	This study
BCL2 F	CTG CAC CTG ACG CCC TTC ACC	This study
BCL2 R	GGG CCA AAC TGA GCA GAG TC	This study
BIRC3 F	GTT CTC TGA CCC AAC CCA GA	Yang et al, 2016 [1]
BIRC3 R	GAG CAA TTG TTG GCT GAT GA	Yang et al, 2016 [1]
BIRC5 F	GGA CCA CCG CAT CTC TAC AT	This study
BIRC5 R	GAC CTT TCT TCG CAG TTT CG	This study
CA9 F	CTG GTG ACT TCG GCT ACA GC	Jamali et al, 2015 [2]
CA9 R	CTA GGA TGA CAC CAG CAG CCA G	Jamali et al, 2015 [2]
FOXM1 F	TGC AGC TAG GGA TGT GAA TCT TC	This study
FOXM1 R	GGA GCC CAG TCC ATC AGA ACT	This study
GAPDH F	GAC AGT CAG CCG CAT CTT C	This study
GAPDH R	CGT TGA CTC CGA CCT TCA C	This study
HIF1A F	GAA AGC GCA AGT CTT CAA AG	This study
HIF1A R	TGG GTA GGA GAT GGA GAT GC	This study
IL24 F	AAG CCT GTG GAC TTT AGC CAG AC	Li et al, 2015 [3]
IL24 R	GCA CTC GTG ATG TTA TCC TGA GC	Li et al, 2015 [3]
PDCD4 F	AGT GAC GCC CTT AGA AGT GG	This study
PDCD4 R	TCA TAT CCA CCT CCT CCA CA	This study
TNFA F	GCC CAG GCA GTC AGA TCA TCT	Ramakrishnan et al, 2011 [4]
TNFA R	TTG AGG GTT TGC TAC AAC ATG G	Ramakrishnan et al, 2011 [4]
TNFSF15 F	CAC CTC TTA GAG CAG ACG GAG AT	Migone et al, 2002 [5]
TNFSF15 R	TTA AAG TGC TGT GTG GGA GTT TG	Migone et al, 2003 [5]

REFERENCES

1. Yang Y, Kelly P, Shaffer AL 3rd, Schmitz R, Yoo HM, Liu X, Huang da W, Webster D, Young RM, Nakagawa M, Ceribelli M, Wright GW, Yang Y, et al. Targeting Non-proteolytic Protein Ubiquitination for the Treatment of Diffuse Large B Cell Lymphoma. Cancer Cell. 2016; 29:494–507.
2. Jamali S, Klier M, Ames S, Barros LF, McKenna R, Deitmer JW, Becker HM. Hypoxia-induced carbonic anhydrase IX facilitates lactate flux in human breast cancer cells by non-catalytic function. Sci Rep. 2015; 5:13605.
3. Li J, Yang D, Wang W, Piao S, Zhou J, Saiyin W, Zheng C, Sun H, Li Y. Inhibition of autophagy by 3-MA enhances IL-24-induced apoptosis in human oral squamous cell carcinoma cells. J Exp Clin Cancer Res. 2015; 34:97.
4. Ramakrishnan P, Baltimore D. Sam68 is required for both NF-kappaB activation and apoptosis signaling by the TNF receptor. Mol Cell. 2011; 43:167–179.
5. Migone TS, Zhang J, Luo X, Zhuang L, Chen C, Hu B, Hong JS, Perry JW, Chen SF, Zhou JX, Cho YH, Ullrich S, Kanakaraj P, et al. TL1A is a TNF-like ligand for DR3 and TR6/DcR3 and functions as a T cell costimulator. Immunity. 2002; 16:479–492.
