# Supplementary material for: Targeting of apoptotic pathways by SMAC or BH3 mimetics distinctly sensitizes paclitaxel-resistant triple negative breast cancer cells
Source: Oncotarget. 2017 Feb 6;8(28):45088–104. doi: 10.18632/oncotarget.15125 (PMC5542169; doi:10.18632/oncotarget.15125)
Supplement: Supplementary file 2 [file oncotarget-08-45088-s002.docx]

**Supplementary Table 1**: Complete list of the 320 small molecule compounds employed in the High-Throughput Screen.

| **Compound Name** | **Selleck Catalog Number** | **Compound Properties/Target** |
| --- | --- | --- |
| (+)-JQ1 | S7110 | Epigenetic Reader Domain, BET |
| (S)-crizotinib | S7505 | MTH1 (NUDT1) |
| 10058-F4 | S7153 | c-Myc, inhibitsc-Myc-Max interaction and prevents transactivation of c-Myc target gene expression |
| 17-AAG (Tanespimycin) | S1141 | HSP90 (100-fold affinity for cancer cells) |
| 2-Methoxyestradiol | S1233 | HIFa nuclear accumulation and transcriptional activity, Aromatase, depolymerization of microtubules |
| 3-deazaneplanocin A (DZNeP) HCl | S7120 | Histone Methyltransferase (competitive inhibitor of S-adenosylhomocysteine hydrolase, adenosine analog) |
| 4E1RCat | S7370 | ELF4 (dual inhibitor of eIF4E:eIF4G and eIF4E:4E-BP1 interaction; inhibits the binding of eIF4G to eIF4E) |
| 6H05 | S7330 | Rho (allosteric inhibitor of oncogenic K-Ras (G12C) |
| A-769662 | S2697 | AMPK reversible activator |
| Abitrexate (Methotrexate) | S8048 | DHFR (antimetabolite and antifolate drug, which acts by inhibiting the metabolism of folic acid) |
| ABT-199 (GDC-0199 ) | S1001 | BCL-2 (>4800-fold more selective versus Bcl-xL and Bcl-w, no activity to Mcl-1) |
| ABT-263 (Navitoclax) | S1002 | Bcl-xL, Bcl-2, Bcl-w, binds more weakly to Mcl-1 and A1 |
| ABT-737 | S1802 | Bcl-xL, Bcl-2 and Bcl-w, Autophagy; BH3 mimetic; no inhibition observed against Mcl-1, Bcl-B or Bfl-1 |
| Afatinib (BIBW2992) | S1011 | EGFR,HER2 (including EGFR(wt), EGFR(L858R), EGFR(L858R/T790M); 100-fold more active against Gefitinib-resistant L858R-T790M EGFR mutant |
| AG-1478 (Tyrphostin AG-1478) | S2728 | EGFR (almost no activity on HER2-Neu, PDGFR, Trk, Bcr-Abl and InsR) |
| AGI-6780 | S7241 | IDH2 (IDH2 R140Q mutant) |
| AICAR (Acadesine) | S1133 | AMPK activator (causes accumulation of ZMP, which mimics the stimulating effect of AMP on AMPK and AMPK kinase) |
| Aliskiren Hemifumarate | S2199 | RAAS (renin inhibitor) |
| Allopurinol (Zyloprim) | S1630 | OX Receptor (xanthine oxidase) |
| Almorexant HCl | S2160 | OX receptor (orally active, dual orexin receptor 1 and 2 antagonist) |
| Alogliptin£¨SYR-322£© | S2868 | DPP-4 (>10,000-fold selectivity over DPP-8 and DPP-9) |
| Alpelisib (BYL719) | S2814 | PI3Ka (minimal effect on PI3Kβ/γ/δ) |
| AMG-073 HCl (Cinacalcet hydrochloride) | S1260 | CaSR (new class of compounds for the treatment of hyperparathyroidism) |
| Amprolium HCl | S4144 | Thiamin antagonist; prevents carbohydrate synthesis by blocking thiamine uptake |
| Anastrozole | S1188 | Aromatase (3rd-generation nonsteroidal selective) |
| Apatinib (YN968D1) | S2221 | VEGFR2 |
| Apigenin | S2262 | P450 (CYP2C9) |
| Apoptosis Activator 2 | S2927 | Caspase 3 activation, PARP cleavage, DNA fragmentation, leads to Apaf-1 dependent destruction of cells; inactive to HMEC, PREC, MCF-10A |
| Aprepitant (MK-0869) | S1189 | Substance P (neurokinin-1 Receptor antagonist) |
| ARQ 621 | S7355 | Kinesin (Eg5 mitotic motor protein inhibitor) |
| Atglistatin | S7364 | ATGL (adipose triglyceride lipase) |
| Atorvastatin calcium (Lipitor) | S2077 | HMG-CoA Reductase (cholesterol-lowering medication that blocks the production of cholesterol) |
| AZ20 | S7050 | ATM/ATR (8-fold selectivity over mTOR) |
| Azacitidine (Vidaza) | S1782 | DNA Methyltransferase (nucleoside analogue of cytidine that specifically inhibits DNA methylation by trapping DNA methyltransferases) |
| Azaguanine-8 | S4194 | Purine analog showing antineoplastic activity by competing with guanine in the metabolism |
| AZD1208 | S7104 | Pim (1/2/3) |
| AZD2461 | S7029 | PARP (with low affinity for Pgp than Olaparib) |
| AZD6244 (Selumetinib) |  | MEK1 (also inhibits ERK1/2 phosphorylation; no inhibition to p38α, MKK6, EGFR, ErbB2, ERK2, B-Raf) |
| AZD7545 | S7517 | PDHK |
| AZD7762 | S1532 | Chk (1/2) (less potent against CAM, Yes, Fyn, Lyn, Hck and Lck) |
| AZD8055 | S1555 | mTOR (excellent selectivity (∼1,000-fold) against PI3K isoforms and ATM/DNA-PK) |
| Barasertib (AZD1152-HQPA) | S1147 | Aurora B (~100 fold more selective over Aurora A) |
| Bardoxolone Methyl | S8078 | IκB/IKK (showing potent proapoptotic and anti-inflammatory activities. Phase 3. Activator of the Nrf2 pathway) |
| BAY 11-7082 (BAY 11-7821) | S2913 | IκB/IKK,E2 conjugating; NF-κB inhibitor, inhibits TNFα-induced IκBα phosphorylation |
| Betahistine 2HCl | S3176 | Histamine H3 Receptor |
| BI 2536 | S1109 | PLK (mainly 1) |
| BIBR1532 | S1186 | Telomerase |
| BI-D1870 | S2843 | S6 Kinase |
| Birinapant (TL32711) | S7015 | IAP (SMAC mimetic antagonist, mostly to cIAP1, less potent to XIAP) |
| BIX 02189 | S1531 | MEK5 (also inhibits ERK5 catalytic activity; it does not inhibit closely related kinases MEK1, MEK2, ERK2, and JNK2) |
| BKM120 (NVP-BKM120, Buparlisib) | S2247 | PI3K (p110α/β/δ/γ. Reduced potency against VPS34, mTOR, DNAPK, little activity to PI4Kβ) |
| BMS 777607 | S1561 | Axl, c-Met (+Ron, Tyro3) |
| BMS-708163 (Avagacestat) |  | Gamma-secretase (Aβ40, Aβ42), Beta Amyloid (193-fold selectivity against Notch) |
| BMS-833923 (XL139) | S7138 | Smoothened/Hedgehog (antagonist) |
| Bortezomib (Velcade) | S1013 | Proteasome (20S) |
| Bosentan | S4220 | Endothelin Receptor (antagonist for ET-A and ET-B) |
| Bosutinib (SKI-606) | S1014 | Src (dual Src/Abl) |
| Brefeldin A | S7046 | ATPase, Autophagy (lactone antibiotic, ATPase inhibitor for protein transport, induces cancer cell differentiation and apoptosis) |
| Brivanib (BMS-540215) | S1084 | FGFR,VEGFR2 (moderate potency against VEGFR-1 and FGFR-1, >240-fold against PDGFR-β) |
| BTB06584 | S7460 | Fo-ATPase (IF1-dependent, selective inhibitor of the mitochondrial F1 Fo-ATPase) |
| Bufexamac | S3023 | COX (for IFN-α release) |
| Bupivacaine hydrochloride (Marcain) | S2454 | cAMP inhibitor |
| BV-6 | S7597 | IAP (SMAC mimetic, dual cIAP and XIAP inhibitor) |
| C646 | S7152 | Histone Acetyltransferase, p300 |
| Calcitriol (Rocaltrol) | S1466 | Vitamin D active form (it is) |
| Camptothecin | S1288 | Topoisomerase I (Cytotoxic quinoline alkaloid) |
| Candesartan (Atacand) | S1578 | RAAS (angiotensin II type 1 (AT1R) receptor antagonist) |
| Capecitabine (Xeloda) | S1156 | DNA/RNA Synthesis (tumor-selective fluoropyrimidine carbamate which achieves higher intratumoral 5-FU level with lower toxicity than 5-FU) |
| Carbamazepine (Carbatrol) | S1693 | Autophagy,Sodium Channel |
| Carfilzomib (PR-171) | S2853 | Proteasome (preferential in vitro inhibitory potency against the ChT-L activity in the β5 subunit, little or no effect on the PGPH and T-L activities) |
| Carmofur | S1289 | DNA/RNA Synthesis (pyrimidine analogue) |
| CC-292 (AVL-292) |  | BTK |
| Celecoxib | S1261 | COX |
| Cepharanthine | S4238 | TNF-α-mediated NFκB stimulation inhibition, plasma membrane lipid peroxidation, platelet aggregation; suppression of cytokine production; AMPK induction |
| Ceritinib (LDK378) |  | ALK (40- and 35-fold selectivity against IGF-1R and InsR) |
| CFTRinh 172 | S7139 | CFTR (voltage-independent, selective CFTR inhibitor; no effects on MDR1, ATP-sensitive K+ channels, or other transporters) |
| CGP 57380 | S7421 | MNK (no inhibitory activity on p38, JNK1, ERK1 and -2, PKC, or c-Src-like kinases) |
| CGS 21680 HCl | S2153 | 5-alpha Reductase (adenosine A2 receptor agonist) |
| CH5138303 | S7340 | HSP90 (orally available) |
| CHIR-124 | S2683 | Chk1 (2,000-fold selectivity against Chk2, 500- to 5,000-fold less activity against CDK2/4 and Cdc2) |
| CHIR-99021 (CT99021) HCl | S2924 | GSK-3a/b (distinguishes between GSK-3 and its closest homologs Cdc2 and ERK2) |
| Chlorprothixene | S1771 | Dopamine Receptor |
| Ciclopirox ethanolamine | S3019 | ATPase (broad-spectrum antifungal agent working as an iron chelator) |
| Cilengitide (EMD 121974, NSC 707544) | S7077 | Integrin (inhibitor for αvβ3 and αvβ5 receptors) |
| Cilostazol | S1294 | PDE and adenosine uptake inhibitor |
| CK-636 | S7497 | Arp2/3 (inhibition of actin polymerization) |
| Cobicistat (GS-9350) | S2900 | P450 (CYP3A) |
| CP 673451 | S1536 | PDGFRα/β (>450-fold selectivity over other angiogenic receptors; antiangiogenic and antitumor activity) |
| CP-91149 | S2717 | Phosphorylase (glycogen phosphorylase (GP) inhibitor more potent in the presence of glucose) |
| CPI-613 | S2776 | Dehydrogenase (α-ketoglutarate); disrupts mitochondrial metabolism |
| Crizotinib (PF-02341066) | S1068 | c-Met, ALK |
| CRT0044876 | S7449 | APE |
| Cryptotanshinone | S2285 | STAT3 |
| CTEP (RO4956371) | S2861 | GluR (mGlu5 receptor) |
| CYT997 (Lexibulin) | S2195 | Microtubule polymerization |
| Danusertib (PHA-739358) | S1107 | Aurora Kinase A/B/C (modestly potent to Abl, TrkA, c-RET and FGFR1, and less potent to Lck, VEGFR2/3, c-Kit, CDK2) |
| Darapladib (SB-480848) | S7520 | Phospholipase (lipoprotein-associated phospholipase A2 (Lp-PLA2) |
| Dasatinib (BMS-354825) | S1021 | Src, Abl, c-Kit |
| Decamethonium Bromide | S4072 | AChR (nicotinic AChR partial agonist and neuromuscular blocking agent) |
| Desloratadine | S4012 | Histamine H1 Receptor |
| Dexamethasone (DHAP) | S1322 | Autophagy, IL Receptor (anti-inflammatory and immunosuppressant) |
| Dinaciclib (SCH727965) | S2768 | CDK2, CDK5, CDK1, CDK9. It also blocks thymidine (dThd) DNA incorporation |
| Dovitinib (TKI-258) | S1018 | FLT3/c-Kit, FGFR1/3, VEGFR1-4; less potent to InsR, EGFR, c-Met, EphA2, Tie2, IGF-1R and HER2 |
| Doxorubicin (Adriamycin) | S1208 | Topoisomerase II, Autophagy, induces DNA damage and apoptosis |
| E7080 (Lenvatinib) | S1202 | VEGFR2(KDR)/VEGFR3(Flt-4), less potent against VEGFR1/Flt-1, ~10-fold more selective for VEGFR2/3 against FGFR1, PDGFRα/β |
| EHT 1864 | S7482 | Rho (Rac1, Rac1b, Rac2 and Rac3) |
| Elesclomol | S1052 | HSP; oxidative stress inducer that elicits pro-apoptosis events among tumor cells |
| Empagliflozin (BI10773) | S8022 | SGLT (SGL2; >300-fold selectivity over SGLT-, -4, -5) |
| Entospletinib (GS-9973) | S7523 | Syk (orally bioavailable) |
| Enzastaurin (LY317615) | S1055 | PKCβ (6- to 20-fold selectivity against PKCα, PKCγ and PKCε) |
| EPZ-5676 | S7062 | Histone Methyltransferase (DOT1L) |
| EPZ-6438 (E7438) | S7128 | Histone Methyltransferase (EZH2) |
| Erastin | S7242 | Ferroptosis activator (acting on mitochondrial VDAC, exhibiting selectivity for tumor cells bearing oncogenic RAS) |
| Erlotinib(OSI-744) | S1023 | EGFR, Autophagy |
| Esomeprazole sodium (Nexium) | S2233 | ATPase, Proton pump inhibitor |
| Estriol | S2466 | Estrogen/Progestogen Receptor (Estriol is an antagonist of the G-protein coupled ER in ER-negative breast cancer cells) |
| Etoposide (VP-16) | S1225 | Topoisomerase II (semisynthetic derivative of podophyllotoxin) |
| Evacetrapib (LY2484595) | S2925 | CETP |
| Everolimus (RAD001) | S1120 | mTOR inhibitor of FKBP12 |
| EX 527 (Selisistat) | S1541 | SIRT1 inhibitor; >200-fold selectivity against SIRT2 and SIRT3 |
| Ferrostatin-1 (Fer-1) | S7243 | Ferroptosis inhibitor |
| Fingolimod (FTY720) | S5002 | S1P Receptor (S1P antagonist) |
| FLI-06 | S7399 | Notch |
| Fludarabine (Fludara) | S1491 | STAT1 activation inhibitor and a DNA synthesis inhibitor |
| Flumazenil | S1332 | GABA Receptor (benzodiazepine antagonist) |
| Flutamide (Eulexin) | S1908 | Androgen Receptor |
| Forskolin | S2449 | cAMP inducer |
| Galunisertib (LY2157299) |  | TGF-beta/Smad (TGFβ receptor I (TβRI) inhibitor) |
| Gefitinib (Iressa) | S1025 | EGFR (Tyr1173, Tyr992, Tyr1173) |
| Gemcitabine (Gemzar) | S1714 | Autophagy, DNA/RNA Synthesis; Antimetabolite |
| GF109203X | S7208 | PKCα, PKCβI, PKCβII, and PKCγ; >3000-fold selectivity for PKC as compared to EGFR, PDGFR and insulin receptor |
| Gimeracil | S2055 | Dehydrogenase (dihydropyrimidine); inhibits the early step in homologous recombination for double strand breaks repair |
| Gliquidone | S3151 | Potassium Channel antagonist (ATP sensitive) |
| GM6001 (Ilomastat, Galardin) | S7157 | MMP (MMP-1, MMP-2, MMP-3, MMP-7, MMP-8, MMP-9, MMP-12, MMP-14, and MMP-26) |
| GNE-0877 | S7367 | LRRK2 |
| GSK1120212 (Trametinib) | S2673 | MEK1/2 (no inhibition of the kinase activities of c-Raf, B-Raf, ERK1/2) |
| GSK1904529A | S1093 | IGF-1R, IR |
| GSK2334470 | S7087 | PDK-1 (no activity at other close related AGC-kinases) |
| GSK256066 | S2620 | PDE4B |
| GSK2606414 | S7307 | PERK |
| GSK2636771 | S8002 | PI3Kβ-selective inhibitor, sensitive to PTEN null cell lines |
| GSK2656157 | S7033 | PERK |
| GW0742 | S8020 | PPARβ/δ agonist, 1000-fold selectivity over hPPARα and hPPARγ |
| GW2580 | S8042 | CSF-1R |
| GW-842166X | S2778 | Cannabinoid Receptor |
| H 89 2HCl | S1582 | PKA |
| HO-3867 | S7501 | STAT3 |
| Icotinib | S2922 | EGFR |
| Imatinib (STI571) | S2475 | PDGFR, c-Kit v-Abl, c-Kit |
| Imatinib Mesylate | S1026 | PDGFR, c-Kit v-Abl, c-Kit (orally bioavailable) |
| IMD0354 | S2864 | IKKβ inhibitor, blocks IκBα phosphorylation in NF-κB pathway |
| INCB024360 | S7587 | IDO1 |
| INH6 | S7494 | Microtubules; Hec1 inhibitor, disrupts the Hec1/Nek2 interaction and causes chromosome mis-alignment |
| Iniparib (BSI-201) | S1087 | PARP1, effective in TNBC |
| IOX2 | S2919 | HIF1a via inhibition of HIF-1α prolyl hydroxylase-2 (PHD2) >100-fold selectivity over JMJD2A, JMJD2C, JMJD2E, JMJD3, or the 2OG oxygenase FIH |
| IPA-3 | S7093 | PAK1 (no inhibition of PAK 4-6) |
| Ipatasertib (GDC-0068) | S2808 | Akt1/2/3 (620-fold selectivity over PKA. |
| Ispinesib (SB-715992) | S1452 | Kinesin |
| IWR-1-endo | S7086 | Wnt pathway inhibitor; induces Axin2 protein; promotes β-catenin phosphorylation by stabilizing Axin-scaffolded destruction complexes |
| JNJ 26854165 (Serdemetan) | S1172 | p53, E3 Ligase |
| JNK inhibitor IX | S7508 | JNK |
| JNK-IN-8 | S4901 | JNK1, JNK2, JNK4; >10-fold selectivity against MNK2, Fms; no inhibition to c-Kit, Met, PDGFRβ |
| JSH-23 | S7351 | NF-κB transcriptional activity |
| K02288 | S7359 | BMP receptor |
| Ki16425 | S1315 | LPA Receptor |
| KPT-276 | S7251 | CRM1 |
| KU-60019 | S1570 | ATM/ATR (270- and 1600-fold more selective for ATM than DNA-PK and ATR; highly effective radiosensitizer) |
| KX2-391 | S2700 | Src |
| Lapatinib Ditosylate (Tykerb) | S1028 | EGFR, HER2 |
| LDC000067 | S7461 | CDK9 (55/125/210/ >227/ >227-fold selectivity over CDK2/1/4/6/7) |
| LDE225 (NVP-LDE225, Erismodegib) | S2151 | Smoothened antagonist; Hedgehog inhibition |
| LDN-212854 | S7147 | BMP receptor (ALK2, about 2-, 66-, 1641-, and 7135-fold selectivity over ALK1, ALK3, ALK4, and ALK5) |
| LDN-57444 | S7135 | DUB; proteasome inhibitor for Uch-L1 |
| Lenalidomide | S1029 | TNF-α secretion inhibitor |
| Lomeguatrib | S8056 | DNA Methyltransferase (O6-alkylguanine-DNA-alkyltransferase) |
| Losmapimod | S7215 | p38α/β MAPK |
| Lovastatin (Mevacor) | S2061 | HMG-CoA Reductase (hypolipidemic agent) |
| LX1606 hippurate (Telotristat Etiprate) | S2173 | Tryptophan hydroxylase (TPH) inhibitor with potential antiserotonergic activity |
| LY2835219 | S7158 | CDK4, CDK6 |
| LY294002 | S1105 | PI3Kα/δ/β, Autophagy (autophagosome formation) |
| LY335979 (Zosuquidar trihydrochloride) | S1481 | P-gp |
| LY411575 | S2714 | Gamma-secretase; also inhibits Notch clevage |
| Manidipine dihydrochloride (CV-4093) | S2482 | Calcium Channel blocker; used clinically as an antihypertensive |
| Maraviroc | S2003 | CCR5 antagonist for MIP-1α, MIP-1β and RANTES |
| Mdivi-1 | S7162 | Dynamin (selective cell-permeable inhibitor of mitochondrial division DRP1 (dynamin-related GTPase) and mitochondrial division Dynamin I (Dnm1) |
| Melatonin | S1204 | Hormone produced in the brain by the pineal gland from the amino acid tryptophan |
| Methazolamide | S4039 | DHFR; antimetabolite and antifolate drug, acts by inhibiting the metabolism of folic acid |
| MG-132 | S2619 | Proteasome; also inhibits calpain (IC50 100 nM and 1.2 uM respectively) |
| MK-1775 | S1525 | Wee1; hinders G2 DNA damage checkpoint |
| MK-2206 dihydrochloride | S1078 | Akt1/2/3 (no other PTKs are inhibited) |
| MK-8776 (SCH 900776) | S2735 | Chk1 (500-fold selectivity against Chk2), CDK |
| ML130 | S2863 | NOD1; inhibits NF-κB activation; 36-fold selectivity over NOD2 |
| ML323 | S7529 | DUB (selective USP1-UAF1 inhibitor) |
| MLN8237 (Alisertib) | S1133 | Aurora Kinase A (>200-fold higher selectivity than Aurora B) |
| MRS 2578 | S2855 | P2 Receptor (P2Y6 receptor antagonist; insignificant activity at P2Y1, P2Y2, P2Y4,and P2Y11) |
| Naltrexone HCl | S2103 | Opioid Receptor |
| Nebivolol | S1549 | Adrenergic β1 Receptor |
| Necrostatin-1 | S8037 | RIP1 inhibitor; inhibits TNF-α-induced necroptosis |
| NH125 | S7436 | ELF2 (eEF-2 kinase inhibitor; >125-fold selectivity over PKC, PKA, CaMKII; potent histidine kinase inhibitor) |
| Nicorandil (Ikorel) | S1971 | Potassium Channel activator |
| Nilotinib (AMN-107) | S1033 | Bcr-Abl (IC50<30 nM) |
| Nimodipine (Nimotop) | S1747 | Calcium Channel, Autophagy (decreases intracellular free Ca2+,Beclin-1 and autophagy) |
| NMDA (N-Methyl-D-aspartic acid) | S7072 | GluR, NMDA receptor |
| NMS-873 | S7285 | p97 |
| NPS-2143 (SB262470) | S2633 | CaSR (Ca(2+) receptor; implicated with PTL resistance) |
| NSC 23766 | S8031 | Rac |
| NSC 405020 | S8072 | MMP (MT1-MMP) |
| NSC319726 | S7149 | p53 (R175) mutant reactivator |
| NSC697923 | S7142 | E2 (inhibitor of the Ub-conjugating enzyme (E2) complex Ubc13-Uev1A) |
| Nutlin-3 | S1061 | Mdm2, E3 ligase; stabilizes p73 in p53-deficient cells |
| Nutlin-3a | S8059 | Mdm2/p53 interaction |
| NVP-AEW541 | S1034 | IGF-1R (27-fold greater selectivity for IGF-1R than InsR) |
| NVP-BHG712 | S2202 | EphB4 (ephrin receptor) inhibitor; discriminates between VEGFR and EphB4 inhibition; activity against c-Raf, c-Src, c-Abl |
| NVP-TAE226 | S2820 | FAK (modestly potent to PYK2; ~10- to 100-fold less potent against InsR, IGF-1R, ALK, and c-Met) |
| Odanacatib (MK 0822) | S1115 | Cathepsin K |
| OG-L002 | S7237 | Histone demethylases, LSD1 (36- and 69-fold selectivity over MAO-B and MAO-A) |
| Olaparib (AZD2281) | S1060 | PARP1/2 (300-times less effective against tankyrase-1) |
| OSI-906 (Linsitinib) | S1091 | IGF-1R |
| OSU-03012 (AR-12) | S1106 | PDK-1 |
| OTX015 | S7360 | BET bromodomain inhibitor (BRD2, BRD3, BRD4) |
| Ouabain | S4016 | Sodium/Potassium Channel ATPase |
| PAC-1 | S2738 | Caspase 3 (procaspase-3 activator); Apoptosis |
| Palomid 529 | S2238 | mTORC1, mTORC2 complexes; reduces phosphorylation of pAktS473, pGSK3βS9, and pS6; no effect on pMAPK or pAktT308 |
| Panobinostat | S1030 | HDAC; HSP90 inactivation |
| PD 0332991 (Palbociclib) HCl | S1116 | CDK4/6 |
| PF 573228 | S2013 | FAK (~50- to 250-fold selective for FAK than Pyk2, CDK1/7 and GSK-3β) |
| PF-04620110 | S7192 | Transferase (diglyceride acyltransferase-1 (DGAT1) |
| PF-3758309 | S7094 | PAK4 |
| PF-4708671 | S2163 | S6 Kinase (S6K1 isoform); 400-fold greater selectivity than S6K2; 4 and >20-fold selectivity than MSK1 and RSK1/2 |
| PF-543 | S7177 | S1P Receptor |
| PF-562271 | S2890 | FAK (~10-fold less potent for Pyk2 than FAK and >100-fold selectivity against other protein kinases, except for some CDKs) |
| Phenformin hydrochloride | S2542 | AMPK |
| Pifithrin-α (PFTα) | S2929 | p53, Autophagy |
| PluriSIn #1 (NSC 14613) | S8076 | Dehydrogenase; inhibitor of the stearoyl-coA desaturase 1 (SCD1), which is able to selectively eliminate hPSCs (human pluripotent stem cells |
| Ponatinib (AP24534) | S1490 | Bcr-Abl, PDGFRα, VEGFR2, FGFR1, Src |
| PR-619 | S7130 | DUB (non selective) |
| Procaine (Novocaine) HCl | S4023 | Sodium Channel, NMDA receptor, nAChR; also inhibits 5-HT3 |
| Propranolol HCl | S4076 | Adrenergic β Receptor |
| PRT062607 (P505-15, PRT2607, BIIB057) HCl | S8032 | Syk |
| PTC-209 | S7372 | BMI-1; results in irreversible reduction of cancer-initiating cells |
| PYR-41 | S7129 | E1 Activating (ubiquitin-activating enzyme E1, with no activity at E2) |
| Quizartinib (AC220) | S1526 | FLT3 |
| Ramelteon (TAK-375) | S1259 | MT1/2 Receptor (melatonin receptor agonist) |
| Rapamycin (Sirolimus) | S1039 | mTOR, Autophagy |
| Regorafenib (BAY 73-4506) | S1178 | RET, c-Kit, Raf-1 and VEGFR1, VEGFR2, VEGFR3, PDGFRβ |
| RepSox | S7223 | TGFβ/Smad (TGFβR-1/ALK5 binding) |
| RGFP966 | S7229 | HDAC (HDAC3 inhibitor; > 200-fold selectivity over other HDACs) |
| Rigosertib (ON-01910) | S1362 | PLK1 (30-fold selectivity than PLK2, no activity against PLK3) |
| Rimonabant (SR141716) | S3021 | Cannabinoid Receptor (CB1) |
| RKI-1447 | S7195 | ROCK1 ,ROCK2 |
| Roscovitine (Seliciclib, CYC202) | S1153 | CDK (Cdc2, CDK2 and CDK5; little effect on CDK4/6) |
| Roxadustat (FG-4592) | S1007 | HIF (HIFα prolyl hydroxylase inhibitor, stabilizes HIF-2 and induces EPO production) |
| Rupatadine Fumarate | S3052 | Histamine H1 Receptor and PAFR inhibitor |
| Ruxolitinib (INCB018424) | S1378 | Histamine H1 Receptor and PAFR inhibitor |
| Safinamide Mesylate (FCE28073) | S1472 | MAO |
| Sal003 | S7437 | ELF2 (cell-permeable eIF-2α phosphatase inhibitor) |
| SANT-1 | S7092 | Smoothened antagonist (binds to Smo receptor); Hedgehog inhibition |
| SB-3CT | S7430 | MMP-2, MMP-9 |
| SB590885 | S2220 | Raf (11-fold greater selectivity for B-Raf over c-Raf, no inhibition to other human kinases) |
| SC75741 | S7273 | NF-κB |
| Selinexor (KPT-330) | S7252 | CRM1 |
| Semaxanib (SU5416) | S2845 | VEGFR(Flk-1/KDR inhibitor; 20-fold more selective for VEGFR than PDGFRβ, lack of activity against EGFR, InsR and FGFR |
| SGC-CBP30 | S7256 | Epigenetic Reader Domain (CREBBP/EP300) |
| SH-4-54 | S7337 | STAT3, STAT5 |
| Sildenafil citrate | S1431 | PDE (cyclic guanosine monophosphate (cGMP)-specific phosphodiesterase type 5 (PDE5) |
| Sirtinol | S2804 | SIRT1, SIRT2 (more potent against Sirtuin 2) |
| SMI-4a | S8005 | Pim1 |
| SN-38 | S4908 | Topoisomerase I (active metabolite of CPT-11, inhibits DNA topoisomerase I, DNA synthesis and causes frequent DNA single-strand breaks) |
| SNX-2112 (PF-04928473) | S2639 | HSP90α, HSP90β (uniformly more potent than 17-AAG) |
| Sorafenib | S7397 | Raf-1, B-Raf and VEGFR-2 |
| SRT1720 | S1129 | SIRT1 activator; >230-fold less potent for SIRT2 and SIRT3 |
| SSR128129E (SSR) | S7167 | FGFR1 (not affecting other related RTKs) |
| SU11274 | S1080 | c-Met (no effects on PGDFRβ, EGFR or Tie2) |
| T0070907 | S2871 | PPARγ (>800-fold selectivity over PPARα and PPARδ) |
| T0901317 | S7076 | Liver X Receptor (LXR, FXR agonist) |
| TAK 715 | S2928 | p38 MAPK (28-fold more selective for p38α over p38β, no inhibition to p38γ/δ, JNK1, ERK1, IKKβ, MEKK1 or TAK1) |
| TAK-875 | S2637 | GPR40 agonist (400-fold more potent than oleic acid) |
| Talazoparib (BMN 673) | S7048 | PARP1 and 2 (does not inhibit PARG; highly sensitive to PTEN mutation) |
| TAME (Tosyl-L-Arginine Methyl Ester) | S2225 | E3 Ligase, APC |
| Tariquidar | S8028 | P-gp |
| Tasisulam (LY573636) | S7326 | Caspase activator |
| Temozolomide | S1237 | DNA damage inducer; Autophagy |
| Temsirolimus (CCI-779, NSC 683864) | S1044 | mTOR |
| Thioridazine hydrochloride | S4374 | Antipsychotic. Targets CSCs. Sub- nanomolar affinity for dopamine and α-adrenergic receptors; inhibits full length recombinant MALT1 (IC50 3.43 μM). It inhibits anti-apoptotic NF-κB signaling and elicits toxic effects selectively on MALT1-dependent ABC-DLBCL cells. Additionally, it suppresses tumor growth activity by targeting the PI3K/Akt/mTOR/p70S6K signaling pathway. |
| TIC10 | S7127 | Akt (and ERK) inactivation and subsequently induction of TRAIL through Foxo3a |
| Ticagrelor | S4079 | P2 Receptor (P2Y12 receptor antagonist, also inhibits CYP2C9 and 4-hydroxylation) |
| Tie2 kinase inhibitor | S1577 | Tie-2 (200-fold more potent than p38) |
| Tipifarnib (Zarnestra) | S1453 | Transferase (farnesyltransferase (FTase) inhibitor; anti-proliferative effects in H-ras or N-ras mutant cells |
| Tofacitinib citrate (CP-690550 citrate) | S5001 | JAK3 (20- to 100-fold less potent against JAK2 and JAK1) |
| Tolcapone | S4021 | Transferase (catechol-O-methyl transferase (COMT) |
| Torin 2 | S2817 | mTOR, ATM/ATR |
| TPCA-1 | S2824 | IκB/IKK-2 (exhibits 22-fold selectivity over IKK-1) |
| Tranylcypromine (2-PCPA) HCl | S4246 | Histone demethylases, MAO-A/B |
| Trelagliptin | S7513 | DPP-4 |
| Triapine | S7470 | DNA Synthesis via ribonucleotide reductase inhibition |
| Trichostatin A (TSA) | S1045 | HDAC (HDAC8 is the only known member of the HDAC-family that is not affected by TSA) |
| Turofexorate Isopropyl (XL335) | S2694 | FXR agonist (highly selective versus other nuclear receptors, such as LXR, PPAR, ER) |
| TWS119 | S1590 | GSK-3β inhibitor; capable of inducing neuronal differentiation and may be useful to stem cell biology |
| U-104 | S2866 | Carbonic Anhydrase CA IX and CA XII; very low inhibition for CA I and CA II |
| UK 383367 | S2224 | Procollagen C Proteinase (excellent selectivity over MMPs) |
| UNC1215 | S7088 | Epigenetic Reader Domain, MBT |
| UNC1999 | S7165 | Histone Methyltransferase (EZH1/2) |
| UNC669 | S7373 | MBT |
| URB597 | S2631 | FAAH; no activity on other cannabinoid-related targets |
| VE-821 | S8007 | ATM/ATR |
| Vorinostat (SAHA) | S1047 | HDAC, Autophagy |
| VX-680 (MK-0457, Tozasertib) | S1048 | Aurora Kinase (mostly against Aurora A; less potent towards Aurora B/Aurora C; 100-fold more selective for Aurora A than 55 other kinases) |
| Wnt-C59 (C59) | S7037 | Wnt/beta-catenin |
| WY 14643 (Pirinixic Acid) | S8029 | PPARα |
| WZ 811 | S2912 | CXCR4 antagonist |
| WZ4003 | S7317 | AMPK; NUAK1 and NUAK2 kinase inhibitor, without significant inhibition on 139 other kinases |
| XAV-939 | S1180 | Wnt/beta-catenin |
| XMD8-92 | S7525 | ERK (BMK1/ERK5 inhibitor) |
| Y-27632 2HCl | S1049 | ROCK1 (p160ROCK), Autophagy (>200-fold selectivity over other kinases, including PKC, cAMP-dependent protein kinase, MLCK and PAK) |
| YH239-EE | S7489 | Mdm2 (p53-MDM2 antagonist and an apoptosis inducer. Effective in mut-p53 cell lines as well) |
| YM155 (Sepantronium Bromide) | S1130 | Survivin |
| ZCL 278 | S7293 | Cdc42 GTPase |
| Zebularine | S7113 | DNA Methyltransferase; also inhibits cytidinedeaminase |
| Zibotentan (ZD4054) | S1456 | Endothelin Receptor (ETA); no activity at ETB |
| Z-VAD-FMK | S7023 | Caspase (cell-permeable, irreversible pan-caspase inhibitor, blocks all features of apoptosis) |
